# Mapping the Multiple Health System Responsiveness Mechanisms in One Local Health System: A Scoping Review of the Western Cape Provincial Health System of South Africa

**DOI:** 10.34172/ijhpm.2021.85

**Published:** 2021-08-21

**Authors:** Tammy Sutherns, Jill Olivier

**Affiliations:** Division of Health Policy and Systems, School of Public Health and Medicine, University of Cape Town, Cape Town, South Africa.

**Keywords:** South Africa, Health System, Responsiveness, Accountability, Feedback Mechanism

## Abstract

**Background:** Despite governments striving for responsive health systems and the implementation of mechanisms to foster better citizen feedback and strengthen accountability and stewardship, these mechanisms do not always function in effective, equitable, or efficient ways. There is also limited evidence that maps the diverse array of responsiveness mechanisms coherently across a particular health system, especially in low- and middle-income country (LMIC) contexts.

**Methods:** This scoping review presents a cross-sectional ‘map’ of types of health system responsiveness mechanisms; the regulatory environment; and evidence available about these; and assesses what is known about their functionality in a particular local South African health system; the Western Cape (WC) province. Multiple forms of indexed and grey literature were synthesized to provide a contextualized understanding of current ‘formal’ responsiveness mechanisms mandated in national and provincial policies and guidelines (n = 379). Various forms of secondary analysis were applied across quantitative and qualitative data, including thematic and time-series analyses. An expert checking process was conducted, with three local field experts, as a final step to check the veracity of the analytics and conclusions made.

**Results:** National, provincial and district policies make provision for health system responsiveness, including varied mechanisms intended to foster public feedback. However, while some are shown to be functioning and effective, there are major barriers faced by all, such as resource and capacity constraints, and a lack of clarity about roles and responsibilities. Most mechanisms exist in isolation, failing to feed into an overarching strategy for improved responsiveness.

**Conclusion:** The lack of synergy between mechanisms or analysis of varied forms of feedback is a missed opportunity. Decision-makers are unable to see trends or gaps in the flow of feedback, check whether all voices are heard or fully understand whether/how systemic response occurs. Urgent health system work lies in the research of macro ‘whole’ systems responsiveness (levels, development, trends).

## Background

**Key Messages**

**Implications for policy makers**

- Understanding how mechanisms intended to improve health system responsiveness function together, is critically important for policy development and implementation.
- The gap between policy, policy implementation (and lack of comprehensive monitoring and evaluation), are major factors for why response to public voice often ends up being more aspirational than actual.
- Policy-makers have a role to play in enhancing synergy between multiple mechanisms at different levels of government, so that overarching health system responsiveness can be strengthened.

**Implications for the public**

The goal of health system responsiveness is intended to prioritize public participation in shaping their health systems – well beyond gathering patient feedback to the quality of health services. Mechanisms have been put in place by government, intended to support this, however, their impact on improving health system responsiveness were found to be not optimally functional (at least in this particular case). This study maps these mechanisms across a particular health system (the Western Cape [WC] province of South Africa [SA]), and begins to investigate their functionality and contribution towards health system responsiveness. It is recommended that systems actors (such as civil society groups) and researchers take a more macro ‘whole systems’ perspective, considering how varied forms of feedback is channeled to decision-makers. This will facilitate the monitoring of responsiveness.

In its framing of health systems, the World Health Organization (WHO) has proposed three goals: improved health (level and equity), social and financial risk protection and improved efficiency, and health system responsiveness.^1^ Health system responsiveness can be defined as, “*The extent to which a health provider or health policy-maker demonstrates receptivity to the ideas and concerns raised by citizens by implementing changes to the decision-making or management structure, culture, policies or practices*.”^2^ Responsiveness is closely tied to the broader idea of citizen, community, or public participation in health systems – which is a core ideal, promulgated in the Alma Ata Declaration, and gaining renewed attention, visible in national policies and guidelines across the world.^2-4^

Responsiveness, and the mechanisms that foster it, can lead to health improvements as well as societal and rights advances, including improved health outcomes, patient satisfaction, health service equity, health rights, service utilization, among other benefits.^2,5,6^ The interaction within these mechanisms, and the information that should flow through them are seen as key for health system responsiveness, where the public can provide feedback on experiences and perceptions (such as complaints/compliments/suggestions), and the health system should be receptive to, and utilize this for context-adapted service and systematic improvements.^5^ Health systems are understood to ‘collect’ feedback in multiple ways, such as data collection driven by service-providers, via researchers and manager’s enquiries, or where service users are able/requested to provide feedback through mechanisms such as suggestion boxes, telephone hotlines or surveys.^7^ Globally, two popular mechanisms are patient satisfaction surveys (PSSs) at point of exit^7,8^ and health facility committees.^9^

There is substantial evidence already on community accountability and public participation in health systems, but there is much less on public participation towards health system responsiveness, especially in low- and middle-income countries (LMICs).^4,10^ Part of the challenge is the broad evidential terrain, which crosses over varied terminologies and theoretical framings. For example, the use of the word ‘citizen’ has been challenged in relation to responsiveness, should the system only respond to officially legitimized citizenry?, as has ‘community’ as being too indistinct. Similarly, there is varied work on ‘community voice,’ ‘information,’ and ‘feedback,’ that are distinct fields of enquiry, but all relevant to responsiveness. The under-represented research on public involvement in health system responsiveness awkwardly straddle different academic terrains. In this article, we use the more generic term ‘public,’ and ‘feedback’ as all types of input from patients *and* the broader public (whether receptively acted on by the system or not), and ‘responsiveness mechanisms’ as any measures that channel feedback and support information sharing and communication of patient and public experiences and expectations, monitoring, and mediation.

There is some empirical work evaluating the presence of responsiveness mechanisms in LMICs, but this tends to focus only on a few most commonly implemented and legislated mechanisms, in isolation of each other, and rarely assesses their functioning.^11^ There are many hypotheses, but a lack of explanatory evidence on the link between generating feedback from the public, and how this then leads to service improvement and systems strengthening.^7^ There is insufficient synthesis across mechanism type within a particular system-setting, which prevents actors and researchers from gaining a ‘systems-wide’ understanding of health system responsiveness; or from evaluating changes in systems responsiveness within different systems; or from understanding context-specific factors in relation to responsiveness.^2^ Suitable research and measurement tools for such explorations are also lacking.^6,12^

Most responsiveness mechanisms and the assessment thereof overwhelmingly focus on *patient *feedback.^13^ Yet, a health system and the mechanisms required for receiving and responding to ‘public feedback’ is made up of many actors, including ‘community’ (varied individuals and groups at different levels, including civil society organizations and close-to-community cadres such as ‘community health workers’) and ‘health providers’ (those responsible for facilitating, managing, redressing, and responding to feedback).^14^ Ensuring mechanisms channel feedback from more than patients, and then assessing such mechanisms together as part of a systemic-level assessment rather than in isolation, supports more robust equity assessment, allows for checking of whose voice and expectations are being taken into account and who remains silent, and how contextual factors such as public values, the political climate and gender relations play a role.^2,15-17^

 This scoping review aims to describe the multiple types of responsiveness mechanisms at play in a particular health system, namely, the public health system of the Western Cape (WC) province of South Africa (SA), in order to describe the system and its local policy context to more effectively support efforts towards strengthening health system responsiveness in this local system, and also to assess approaches to assessing system responsiveness.

## Methods

 This scoping review presents a cross-sectional ‘map’ of types of health system responsiveness mechanisms, the evidence available about these, and assesses what is known about their functionality in a particular local South African health system. While this review is not a case study, the WC provincial health system is presented as a ‘case’ of what can be understood by conducting a whole-systems assessment across multiple mechanisms.

 Multiple forms of indexed and grey literature were scrutinized and synthesized to provide a contextualized understanding of current ‘formal’ responsiveness mechanisms that are mandated in national and provincial policies and guidelines.

This scoping review was sequentially mixed methods in nature, with research conducted in phases. In the first phase, an iterative analysis was conducted across varied forms of secondary data (roughly calculated as 379 items included in the review, see Table 1).

**Table 1:** Overview of Data Sources Used

**Data Type**	**Type and NumberAssessed**	**Location**
Secondary literature: peer-reviewed articles	*Quantitative and Qualitative *[Number = 301 assessed, PRISMA showing 134 included (Supplementary file 1)	PubMed, EMBASE, CINHAL, other academic platforms & portals
Secondary literature: institutional reports [showing internal review]	Quantitative and qualitative [Number = 76]	AMREF, ARNOVA, CADRE, CREHS, Centre for Health Policy, Center for Global Development, EQUINET, Global Health Workforce Alliance, Harvard University, Health Systems Trust, International Institute for Labour Studies, Khulamani Support Group, MRC, SADOH, TAC, The Alliance for Health Policy and Systems Research, The Global Fund, The Learning Network, The World Bank, UNDP, UNFPA, UN Global Pulse, USAID, WCDOH, WHO, Zimbabwe Equity Watch
Current or ongoing studies	Quantitative and qualitative [Number = 71]	The NHRD
SA/WC policy documents, including primary materials [eg, forms, posters] and SA National Guideline to Manage Complaints, Compliments, Suggestions in the Public Health Sector of SA (2017)	Quantitative and qualitative [Number = 129] PRISMA: 51 policy docs analyzed (Supplementary file 1)	SADOH, WCDOH, https://www.idealhealthfacility.org.za/, SA National guideline accessible online: https://tinyurl.com/s4s9v6k
Survey data, guides, reports, client/patient satisfaction and complaints guides and reports	Quantitative and qualitative [Number = 29]	Cape Area Panel Study, General Household Survey, Health Stats SA, SA Demographic and Health Survey, World Health Survey, CADRE, SADOH, WCDOH, HST
Media reports	Quantitative [Number = 10]	https://www.news24.com; https://www.timeslive.co.za; http://www.ewn.co.za; https://www.media24.com/newspapers; http://www.national.archives.gov.za; https://www.sabinet.co.za; https://www.newsbank.com; https://www.iol.co.za/capetimes
Theses	Quantitative and qualitative [Number = 8]	University of Cape Town, University of the Witwatersrand

Abbreviations: NHRD, National Health Research Database; WC, Western Cape; SA, South Africa; WHO, World Health Organization; USAID, United States Agency for International Development; UNFPA, United Nations Population Fund; UNDP, United Nations Development Programme; PRISMA, Preferred Reporting Items for Systematic Reviews and Meta-Analyses; SADOH, South African Department of Health; WCDOH, Western Cape Department of Health; AMREF, African Medical and Research Foundation; ARNOVA, Association for Research on Nonprofit Organizations and Voluntary Action; CADRE, The Centre for AIDS Development, Research and Evaluation; CREHS, Consortium for Research on Equitable Health Systems; EQUINET, The Regional Network on Equity in Health in East and Southern Africa; MRC, The Medical Research Council of South Africa; TAC, Treatment Action Campaign; HST, Health System Trust; UN, United Nations. Note: this table reflects relevant data utilized, not all locations searched or materials gathered - see PRISMA diagram for literature review phase, Supplementary file 1.

In addition to published literature, this scoping review extracted and conducted time series analysis across survey data reports, analysed policy documents, and assessed evaluative reports in its second phase. This was similar in nature to the descriptive ‘mapping’ tool proposed for assessment of regulatory policies and processes in LMIC health systems by Sheikh et al.^18^

A large component involved standard review of published materials relating to ‘responsiveness mechanisms in LMIC health systems’ and then in ‘SA and the WC’ more specifically, across several databases, including peer-reviewed journal articles, theses, and internally reviewed institutional reports. The review of LMIC literature contextualized the local evidence, provided the frame for thematic analysis, and substantiated local findings, necessary given the lack of research in this area. The search was limited to English-language materials, published from 2000-2019, although earlier relevant materials identified through trace-searching were included. All materials were assessed for relevance in first round review, and quality in second round review. Supplementary file 1 provides the search terms and variations, PRISMA diagram, and output table (also including as a resource, a more extensive reference list).

The policy review component assessed publicly available content in 75 broader, national South African policy documents and provincial, WC policy documents, with 51 identified as particularly relevant to this study (Supplementary file 2). This included mainly ‘primary’ materials, including the information, education and communication (IEC) materials produced by national and provincial government, and forms, guides and posters related to responsiveness and mechanisms.

 Available survey data from the latest General Household Survey (2018), the Cape Area Panel Study (2012), Health Statistics SA (2019), the SA Demographic and Health survey (2016) and the World Health Survey (2004) was also extracted and compiled. The South African National Health Research Database (NHRD) was reviewed for ongoing studies relating to responsiveness or feedback within SA (we found 79 relevant open studies). Media reports were located via key search terms, which offered further insight into platforms utilized by the public for providing feedback (including the media itself).

 Each collected data-type was assessed for relevance and quality independently, analyzed using an appropriate analytical approach depending on the data type (mainly thematic and time-series analysis), and then synthesized with the other forms of data using a thematically organized extraction sheet and framework, developing a descriptive map (Supplementary files 1 and 2). Data was categorized according to type, national/provincial focus, general responsiveness or individual mechanisms, and broken down by extracting data focus (eg, responsiveness vs community health workers [CHWs]), abstract/summary, publication/source, title and date and first author. This was further categorized into a typology of mechanisms, with responsiveness and functionality assessed for each mechanism (functionality, when data allowed).

 The triangulation across varied data types was an important component of rigor/confirmability in this mixed method review approach. As a further measure to ensure integrity and credibility, the lead researcher (as an actor in the local health system) kept a reflective research diary for observations; regular research team debriefs were held; and joint review of identified materials was conducted (both authors).

 After analysis and draft write-up, a final expert checking process was conducted during 2020 as part of the third phase, in which three local field experts (one academic, one local health system, one from civil society) were asked to assess the draft findings and provide comments, corrections or additions based on their understanding of the local system.

Table 1 depicts the varied publicly available data that was gathered and assessed in sequential phases, including indexed literature, government websites, the National Health Research Committee database, surveys, theses and media sources.

In the next section, we report on findings relating to the regulation of mechanisms in the WC, drawing from synthesized findings from all phases of review (see Supplementary file 1 for a full listing of all resources). We then discuss the main mechanisms types and mechanism functioning.

## Results: Responsiveness Mechanisms in the Western Cape Province

###  Policy Evolution and Context

The WC province is one of nine legislated provinces in SA’s quasi-federal political system, governed by a Premier and currently the Democratic Alliance, the main opposition party to the overall national ruling African National Congress.^19^ Formal mechanisms are detailed in many national, provincial and district policies, plans, guidelines, legislation, annual reports and other documents (Supplementary file 2 and Figure).

**Figure:**
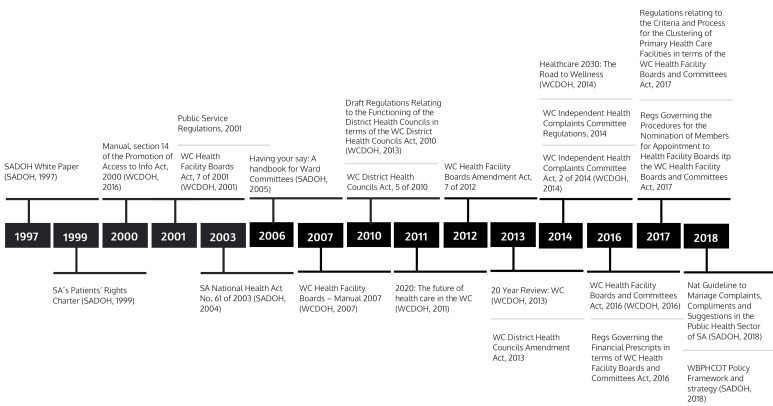
Timeline of Policies/Reports Addressing Mechanisms. Source: authors. Abbreviations: WC, Western Cape; SA, South Africa; SADOH, South African Department of Health; WCDOH, Western Cape Department of Health; WBPHCOT, ward-based primary healthcare outreach team.

Many of these build upon the *National Health Act*, 61 of 2003 (NHA) and the 1999 *Patients’ Rights Charter*, promoting the right for ‘citizens’ to complain and be responded to, and the 1997 *White Paper for the Transformation of the Health System in SA*, whichpromotes decentralization and civil society (NGO, non-governmental organization) collaboration. The *National Guideline to Manage Complaints, Compliments, Suggestions in the Public Health Sector of SA *(*National Guideline)*^20^ is a pivotal guideline relating to responsiveness. Other key mechanisms include the Office of the Health Ombud and WC legislation (Figure). Gathered policy materials also showed (usually) annual reporting on patient satisfaction and complaints data within the national and provincial health systems.

The timeline in Figure depicts how, over time, legislation has narrowed its focus to address mechanisms in isolation from each other – most recently focusing almost exclusively on complaints procedures, ward-based primary healthcare outreach teams (WBPHCOTs), committees, and health facility boards (HFBs). A broader mandated strategy for taking these all into account has not been published on a provincial level since 2014, or at a national level since the 2003 NHA [This information is provided in a more detailed tabular format as Supplementary file 2].

There is therefore provision for health system responsiveness (although sometimes framed in terms of accountability and participation) in national and provincial policy and legislation, and support for the institutionalization of mechanisms for receiving and responding to feedback. The mechanisms visible in the WC (Box 1) can be loosely divided into those that directly support/channel* complaints;* and those that channel feedback in support of *community accountability and participation* processes. The policy documents similarly tend to frame these as different enterprises, even if addressing the same mechanism. Our synthesis suggests there are currently at least 15 distinct mechanism types currently mandated in the province that relate to health system responsiveness.

**Box 1. Formal Mechanisms for Receiving and Responding to Citizen Feedback in the WC**

** Those immediately supporting complaints processes**

- Complaints Committee
- Health Ombud
- ICT mechanisms (hotlines, SMS-hotlines and health information systems)
- Suggestion boxes
- PSSs
- Staff surveys

** Those supporting broader accountability and participation processes**

- District Health Council
- HFBs
- [Health-related] Committees
- CHWs programs
- National Health Council
- NGOs
- Office of Health Standards Compliance

Abbreviations: WC, Western Cape; ICT, Information and Communications Technology; PSSs, patient satisfaction surveys; CHWs, community health workers; HFBs, Health Facility Boards; NGOs, non-governmental organizations.

###  Varied Complaint, Compliment and Suggestion Mechanisms

Complaint/compliment/suggestion processes in the province involve several national and provincial mechanisms, mainly channeling patient feedback and relating to quality assurance strategies. The 2017 *National Guideline* was developed to facilitate information gathering, responsiveness and quality improvements,^20^ – demonstrating the framing of a direct link between complaints processes and health system responsiveness. The SADOH outlines a system to manage complaints (Box 2), showing a flow of information from local to district/provincial to national levels of government, detailing ‘steps’ – although it is widely observed that complaints do not often follow this neat process in reality, for example the common approach of complaint to national media, which goes directly to the national Department of Health, and then flow back ‘down’ the system.

**Box 2. Steps and Stages to Manage Complaints^19^**

Step 1: Enabling complaints

Step 2: Responding to complaints

Step 3. Accountability and learning

** Stage 1:**

- Complaint addressed
- If citizen is not mollified by redressal it is escalated
- If complaint flagged as severe, it is escalated

** Stage 2:**

- Complaint escalated to district or provincial office where it is addressed or further escalated

** Stage 3:**

- Escalation to national public protector, consumer commission, legal system, Health Ombud/OHSC or professional councils and/or boards

Abbreviation: OHSC, Office of Health Standards Compliance.

Mechanisms intended to facilitate these three steps include a health establishment level standard operating practice (SOP), a Complaint, Compliment and Suggestion Committee, standardized feedback forms, complaint/compliment/suggestion boxes, IEC posters or pamphlets (in all official languages), a record system for complaints (including complaint, timeframe and resolution type) and a complaints register.^20^ The process, in terms of the flow of information between levels of government, includes the categorization of formal complaints, which are reported to the Provincial Office, who then submits quarterly reports to the National Office.

This process is detailed but still new, so the degree of integration into the WC health system has not been documented. However, South African Department of Health (SADOH) and Western Cape Department of Health’s (WCDOH’s) annual reports specify how many complaints were received and how many were responded to within 25 working days. No detail is provided on content of complaints or demographic data for those providing complaints, nor is an evaluation of the actual process offered (something requiring a great deal of further investigation). Measures of success in terms of service improvement or health system responsiveness are notably absent. There is particularly little data relating to Step 3 (accountability and learning), the part most relevant to responsiveness, namely, did the system learn from the feedback, and was there a systems-level response beyond individual patient resolution?^14^

*SMS and telephonic hotlines* are also mechanisms facilitating complaints/compliments/suggestions developed in the WC and successfully piloted in 2012, with the plan to expand rollout.^21^ The subsequent year´s annual report makes mention of email, SMS, telephone and ‘Please Call Me’ services displayed on facility notice boards, through which 1984 complaints were reportedly received and 83.2% resolved the following year - with the process supported by anon-profit organization.^22^ Post-2014, WCDOH annual reports and plans no longer make mention of these mechanisms, so it is unclear if further rollout occurred (or at least, it does not seem to be monitored). However, comparing 2015 and 2019 WCDOH website contact information (Supplementary file 3), it is evident that the hotline, SMS and ‘Please Call Me’ numbers still exist and that, in 2019, the Department also offered social media platforms and online forms for public feedback. The backend process including who is responsible for each mechanism, how the feedback is categorized, and who attends to them, remains entirely opaque and unreported.

For maternal health, the national government implemented MomConnect in 2014, a mobile health program for pregnant women. Part of the program is an interactive help desk feature, where expecting mothers are able to provide feedback on both the MomConnect platform and the healthcare facilities that they utilize. A study analyzing MomConnect between 2014 and 2016 showed that 20% to 40% of complaints received via the platform related to the antenatal services that pregnant women received and helped to flag health system issues including drug and vaccine shortages, long wait time and patient abuse or neglect.^23^ The feedback was utilized by health managers at facility-level as well as analyzed at regional and national levels. While an example of a functioning feedback mechanism, this is focused specifically on maternal services and its impact on health system responsiveness is not yet understood, with more data needed.

A three-person Independent *Health Complaints Committee*was appointed in the WC province in 2015, in accordance with the *Western Cape Independent Health Complaints Committee Act, No 2* of 2014. There were media reports about the appointment in 2015 and mention of the committee in the WCDOH 2010/2011, 2012/2013, 2013/2014 and 2014/2015 annual reports, however the current state of the committee is unclear.

Also forming part of the national complaints process is the *Health Ombud*,^24^ who sits in the office of the OHSC (Office of Health Standards Compliance) as an independent complaints investigator. There is an established process for the Health Ombud – which can be utilized once redress has occurred at local levels, reported on annually, however the Ombud flagged in its latest report that it was struggling to fulfil its functions due to resource constraints.^25^

Equity and citizen representation is not a focus in any government reporting in terms of mechanisms. There is not enough data to make detailed conclusions here, but as a very basic assessment, consider that in the *WCDOH Annual Report 2017/2018*,^26^ 6.5 million patients were reported to access services and 5268 complaints were received (91.4% resolved). Therefore, only 0.08% of those accessing services in this period provided feedback in the form of complaints. In terms of hotlines, a 2013 WCDOH press release reported that over a five-month period, 594 complaints were logged (six calls/day average), the majority from one particular Community Health Centre.^21^ During this period, the WCDOH was serving six million people, which means that less than 0.01% utilized the complaints hotline over this period. We do not assess whether this level of complaint-based feedback is appropriate to the quality of care being provided, nor can at this time assess the quality or content of the feedback received, but the basic *quantity* of feedback through these mechanisms appears to be relatively low, considering the size of population utilizing the public health services in the WC, and the known challenges with regards to access and service quality in this LMIC setting.^27^

In SA, there are also 11 official languages, with complaints mechanism information posters available in all 11^28^; but *Patient’s Rights Charter* display materials only available in seven. *The National Guideline *highlights that procedures should be made known to the public in appropriate languages.^20^ Yet, there is no SOP in place for practical application within health clinic contexts (eg, how to decide on the most appropriate languages), nor who is responsible for explaining the procedure to first time users.

###  Overview of Main Mechanism Types

 We provide further description of five main mechanism categories, into which the 15 identified mechanism types identified can be clustered.

####  Patient and Staff Satisfaction Surveys

The WCDOH’s 2030 *Strategy* emphasizes that surveys should be utilized to hear the voice of the patient (at the point of health service utilization), “to provide the basis for ongoing improvements.”^26^ PSSs are conducted annually across facilities in the province and reported in district and provincial annual reports. The satisfaction rate in the latest report was 86%. The WCDOH annual reports show that PSS are used to develop quality improvement plans for issues such as waiting times and staff attitudes at a facility/local level, but further information on this is not detailed or tracked, nor is there evidence on whether issues are endemic or considered across the system. The WC government PSS template was not available for review, however the latest annual report details that R418 000 was spent on conducting the surveys through a consultancy service in 223 provincial facilities with 59 669 surveys captured.^29^ Data on patient satisfaction has also been gathered via non-governmental entities such as researchers or NGOs. Routine and household surveys such as the Cape Area Panel Study, General Household Survey, Health Stats SA, SA Demographic and Health Survey and the World Health Survey do provide information on levels of satisfaction and health outcomes, but no indication was found that any survey data is utilized further by the WCDOH - to gather feedback and generate a systemic response.

The influence of race and socioeconomic status (SES) on perceived quality of care has been explored thoroughly in SA, both being significant predictors of levels of satisfaction – with patients in the ‘white’ race group, and high SES respondents 3.5 times more likely to rank perceived quality of care as ‘excellent’ compared to ‘black’ race groups and low SES respondents in the public health sector.^30,31^ Considering the context and the values outlined in the NHA, improvements in health rights and equity as a measure of success in responsiveness should be a considerable focus for the health system. However, there is no evidence on the role such factors (or other similar factors or vulnerabilities) play in public utilization of mechanisms within the national or provincial health systems,^14^ a seemingly critical missing step. In fact, an evaluation of whether PSSs are representative of the broader public is missing (Box 3). Because of these limitations, the available evidence on the current PSS is unable to support full assessment of changes in system responsiveness.

**Box 3. Are PSS Representative? Findings From the WCDOH Annual Report 2018-2019^28^**

In 2018, 223 facilities in the WC conducted the annual PSS – which means 19% of facilities did not participate. The PSS also excludes midwife obstetrics units, mobile services, psychiatric hospitals, reproductive health facilities and specialized health care facilities. This shows that PSS feedback is not representative of all health services in the WC. Furthermore, while 86% of respondents were satisfied with health services in 2018, it was not clear how the sample of 59 669 patients was selected to participate within the health facilities that were included (and that participants were indeed a representative sample of those accessing health services across SES and race groups).

This is a particular important issue for further consideration, as PSS is often presented in the responsiveness policy and literature as a main mechanism for assessing *systems* responsiveness (not just service level response to a core set of patients already accessing health services).

Abbreviations: PSS, patient satisfaction survey; WC, Western Cape; SES, socioeconomic status.

The WCDOH also promotes *staff satisfaction surveys* and The Barrett Value Survey (conducted every second year), in order to gather feedback from health providers on their experiences and the organizational culture of their health facilities – a mechanism to foster quality improvement plans from within the health service. The WCDOH is responsible for conducting the non-mandatory online staff surveys every second year (last one 2017, then 2020) and publishing results. It is, however, unclear how survey data is utilized for improvements. In 2016, only 38.25% of staff in the WC (who are also citizens in the health system), felt that they received feedback on their suggestions; in 2013 it had been 38.10%; and in 2018, only 35.58% felt their organization was open to employee’s feedback and ideas.^26^ Similar to the identified disconnect between feedback channeled through different mechanisms, so too does there appear to be a lack of synthesis across feedback from different actor groups, including managers, frontline workers, staff, patients and the public.

####  Committees and Health Facility Boards 

Committees and HFBs may sometimes contribute to a complaint/compliment/suggestion process, but have much broader scope, including the planning and provision of services in health facilities.^32^ Health committees are supported by legislation in the form of the *Western Cape Health Facility Boards and Committees Act*, 2016 – made up of no more than 12 members who represent the public served by that primary healthcare (PHC) facility and every hospital should have a HFB (of ≤14 representative members).^33^ A provincial *Facility Board Manual* offers guidelines and highlights the board’s accountability to the public (community, patients and families).^34^ Although formalized in legislation more recently, the *2002-2003 Western Cape Health Annual Report *highlights that HFBs were achieved throughout the province during this period^35^ – showing that there has been a long-standing presence of facility committees, mandated by the NHA.^36^ Both committees and boards are required by the 2016 Act to provide quarterly reports, written reports of activities within the end of each calendar year and measures for cooperation as well as schedule regular meetings.^33^ A database of health committees or HFB meeting minutes or progress reports is not readily accessible, however a record of a meeting held on 17 April 2018 was located, detailing the introduction of the *Western Cape Health Facility Boards and Committee Act*. In 2018, the WCDOH published a call for community members to volunteer for health committees within all the districts.^37^ It is not known how many HFBs or health committees are currently operational in the province since their legislation in 2018. However, in the province, health facilities do need to have a functional clinic committee in place to meet the criteria to be considered an ‘Ideal Clinic,’^26^ and the latest WCDOH annual report reveals that in 2018/2019, 171 facilities achieved ´Ideal Clinic´ status,^26^ which suggests that there are perhaps 171 health facilities in the province reporting functional clinic committees.

####  Community Health Workers

CHWs have garnered a lot of research attention in LMICs, although there is not a lot of formal documentation or legislation on CHWs in the province. A 2014 study conducted in Cape Town showed that CHWs were a critical part of the health workforce, acting as “health educators, advisors, rehabilitation workers and support group facilitators”^38^ and thus can be considered a type of ‘mechanism,’ channeling feedback between system and public.

In SA, there is a lack of formal legislation around CHWs and they are underutilized, despite the post-1994 focus on PHC and the organization of a “highly diverse community care system that evolved around HIV and TB.”^39^ In the WC, NGOs are often responsible for contracting CHWs, although payment may be subsidized through the government, but resourcing, standardized roles and responsibilities, training, supervision, monitoring, financing and governance remain challenges.^39^ Without formalization, it has been argued that CHWs can face deficient working conditions, low pay and poor management.^40^

There have been attempts to formalize, with the *WBPHCOT Policy Framework* launched in 2017,^41^ building on the success of the HIV-engaged CHW programs.^40^ Evidence shows that WBPHCOTs have been operating for a decade, but are not fully-functional, with challenges including “varying perceptions of the CHW roles, lack of knowledge and skills and lack of stakeholders and community support.”^42^ A 2017 review shows that there are only 3275 WBPHCOTs submitting information through the District Health Information Software - 42% of the 7800 mandated.^40^

####  Non-governmental Organizations 

NGOs and civil society do not feature heavily in legislation, only mentioned briefly in the 1997 *White Paper* as having an important role in the delivery and management of health services.^32^ While there are over 50 NGOs listed on the provincial directory of non-profits and civil society organizations^43^ and over 100 000 registered in the country,^44^ their role in terms of strengthening health system responsiveness has not been formalized. Yet, it has been widely observed that civil society and NGOs play a significant advocacy role with regards to patient rights and access in SA and the province, such as their role in advocacy and community mobilization during the height of the HIV/AIDs epidemic, demonstrating the potential for serving as responsiveness mechanisms.^45^ A current challenge is that formalization between civil society and government “may direct funding away from health-related non-profit organizations and in other ways limit their ability to respond independently and critically to the interests of marginalized communities.”^46^

####  “Informal” Feedback Channels

Media also functions as a type of ‘feedback channel’ or mechanism (in its broader sense). Around 2001, the media was a channel for public and civil society advocacy, putting pressure on the government to provide antiretroviral therapy.^47^ There are multiple other examples at a provincial level of the public taking up media-based advocacy – in particular publishing complaints about poor services, which usually gains a secondary media-based response from the WCDOH (or national) authorities.^48^ An analysis of print media coverage of PHC and related research evidence in SA found that over a 16-year period, the WC featured the highest amount of coverage in terms of accountability of the state sectors, with the following topics covered in print media: availability of care (30%), timely access to care (18.5%), culturally appropriate care (1.7%) and package of care (9.8%).^49^ This study also noted that over the 16-year period, 12% of print media coverage related to strike or protest action – a common occurrence in SA, and another way feedback is expressed.^49^ For example, 2007, 2009 and 2010, saw health provider strikes, including violent strikes among nurses, in protest to low pay and work conditions.^50^ There are also frequent reports of community members burning down health facilities (also a form of vandalism) – for example, ‘*Burning down of clinics will only chase away health workers.’*^51^ In 2016, 66.84% of health providers in the province reported experiencing verbal and/or physical abuse from patients in the last year^26^– another area that should be explored in terms of informal forms of feedback and response.

###  Evidence Gaps on Mechanism Functioning and Response to Feedback

The above demonstrates that there are multiple avenues legislated for the public to provide feedback, voice their perceptions and experiences, and potentially support system responsiveness. However, when seeking out routine and evaluative data on the current *functioning* of these legislated mechanisms, we found massive evidence gaps, suggesting that much lip service is paid to responsiveness mechanisms in policy, but this might remain poorly implemented in practice (Table 2). For example, we searched for and reviewed multiple forms of evidence, seeking key elements such as roles and responsibilities, cost evaluations, access information, proof of representation, evaluation of functionality, systems receptivity to feedback, and anything relating to systems response. We felt it was important to publish this effort and evidence gap as a finding, so as to highlight the importance of further empirical research. Table 2 highlights what basic elements are currently unknown, and this was substantiated in our expert checking process, with experts unable to clarify where such evidence could be found. If such data were gathered and analyzed, this would allow for an evaluation of functionality and sustainability of these mechanisms and identify gaps, including missing voices or inequitable access to these mechanisms. It would also allow for assessment of change over time, as we would also expect responsiveness to change over time in a complex adaptive system, and it would be important to track that.^15^

**Table 2:** Mechanisms in the WC, Summary of Missing Data on Functionality

**Mechanism**	**Missing Data on Functionality**
Complaints process	Person/people responsible for investigating, collating feedback, responding to feedback, escalating to next level of government Person/people responsible for addressing complaint on each level of govt before Ombud/Boards Cost/resources needed
Facility complaint feedback form	Person/people responsible for disseminating form Criteria for who receives a form, in which facilities, barriers How is data utilized in responsiveness
Suggestion boxes	Person/people responsible for emptying boxes, investigating, collating feedback How many available in how many facilities, barriers How is feedback data utilized in responsiveness
IEC posters/ pamphlets detailing feedback process	Person/people responsible for putting up posters, distributing pamphlets How many available in how many facilities
Complaints register	Person responsible for filling out, filing, barriers How is data utilized in responsiveness
Complaints Committee	Committee members, process, structure Who do committee members represent Meeting frequency, agenda, barriers Cost/resources needed
SMS/telephone hotline and hotline information	Person/people responsible for answering phone/texts, investigating, collating feedback, Person responsible for distributing information on hotline, which facilities, how often, barriers How is feedback data utilized in responsiveness Cost/resources needed
Health Ombud	How is feedback data utilized in responsiveness Barriers
PSS	Person responsible for distributing, to who How does consultancy ensure equity across respondents, facilities, barriers How is feedback data utilized in responsiveness
Staff satisfaction surveys	Person responsible for distributing, to who, barriers How is feedback data utilized in responsiveness Cost/resources needed
Committees and HFBs	Does each health facility have an operational HFB Does each PHC facility have a health committee Reports of activities, measures for cooperation, records of attendance, minutes, resolutions Role/process for facilitating feedback, how is it utilized in responsiveness, barriers Cost/resources needed
CHWs	How many WBHCOT/CHWs are in operation, where, SOP Role/process for facilitating feedback, how is it utilized in responsiveness, barriers Cost/resources needed
NGOs	Role/process for facilitating feedback, SOP How is it utilized in responsiveness, barriers Cost/resources needed

Abbreviations: WC, Western Cape; IEC, information, education and communication; PSS, patient satisfaction survey; PHC, Primary health care; HFB, health facility board; CHWs, community health workers; SOP, standard operating practice; NGOs, non-governmental organizations; WBHCOT, Ward-based Primary Health Care Outreach Teams.

When functioning as intended, mechanisms should support improved services, as well as *system* responsiveness, and eventually support improvements in health outcomes.^2,5,6^ Yet, this case shows that while policy documentation makes mention of mechanisms, and occasionally details implementation processes, a critical step appears to be missing: *ensuring the health system takes feedback into account* (whether it is mandated to do so), that is, whether system strengthening improvements are made as a result. Without adequate evaluation and monitoring of feedback and the functioning of these mechanisms, and the actions (or inactions) if system actors as a result, the current ‘map’ remains descriptive rather than explanatory, and there remain great unknowns about how and which feedback is utilized for what decision-making, and whether systems’ actors respond then in a way that strengthens the system over time.

 In this findings section we compartmentalized each mechanism for descriptive purposes, however, it becomes apparent that this is how these mechanisms are framed and function in the health system as well – and this is a major barrier to improving system responsiveness, or developing effective learning systems. For example, this review shows that different actors play a role in the various mechanisms, at different levels and there does not seem to be any single ‘hub’ through which feedback is channeled and response coordinated. Since the routinised data that does exist and is reported annually, such as patient and staff satisfaction surveys and complaints received, is not synthesized with data from other mechanisms, such as suggestion boxes, hotlines, legislated cases, committees, surveys, systemic issues are not clearly identifiable. For example, there are wide reports of complaints about waiting times across the gathered literature coming in via varied mechanisms, but they appear to be handled/resolved on an individual basis (usually at patient-level), at particular points in the system; and community-level voices or suggestions appear to get ‘lost’ as it gets translated between levels. The lack of synthesis across feedback streams and through multiple, disconnected mechanism types, therefore means that health system actors are unable to see patterns and trends across the feedback, which does not support whole-of-systems evidence-based decision-making, or a full understanding of the priorities of the public.

The reviewed literature also strongly suggests that feedback-data gets distilled and continuously (re)summarized as it moves via the initial mechanism ‘up’ the system to provincial and then national levels – meaning that upper level decision-makers, are likely only to receive a subjectively filtered ‘sample’ of feedback on which to make macro systems decisions. Furthermore, the feedback loops between community, sub-district, district and national levels are not clearly indicated or mandated. Our review suggests that the ‘response’ part of the feedback loop is often absent, however we say this with caution, as an absence of evidence does not necessarily mean an absence of action. While there is some data of feedback loops in relation to complaints processes, especially the litigation-level complaints that are treated most seriously, there is almost none on informal feedback such as that channeled via media reports or protests, which can be argued to be very ‘loud’ forms of feedback, that should be factored into our understanding of system responsiveness (see Supplementary file 4 for full references relating to each mechanism).

## Discussion

The findings show that there are several mechanisms in place in the provincial and national policy, but implementation is underreported and appears to be more limited, and importantly there is a clear disconnect between mechanisms. The consequences of this can be deadly. In SA, a recent tragedy involving the Life Esidimeni patients (Gauteng Mental Health Marathon Project), is a poignant example of this disconnection, played out in the Gauteng province, where 144 mental health patients died after being transferred from long-stay residential facilities to “under-regulated and unlicensed facilities”^52^ after the failure of a series of mechanisms. The Health Ombud’s findings of the subsequent investigation, found that all of the patients who died at the NGO facilities (95%), did so under “unlawful’ circumstances.”^53^ “While it has exposed major deficiencies in both governance and management, it has also focused attention on the gap between policy and implementation, and between intentions and consequences.”^52^ This example is not anomalous. In the WC many of the formally legislated mechanisms evidence a policy-implementation gap. For example, while complaints processes outline detailed procedures, they do not go further to the ‘systemic response or change’ that is legislated. Another example is health committees, which are now heavily legislated to be part of facility governance, but many studies have shown the challenges of implementation, with many committees present ‘on paper,’ but not functioning according to their mandate.^54^ A 2010 study concluded that community health committees felt “their inputs were neither valued nor considered in the planning and provision of health services.”^55^ These gaps highlight three main areas of consideration when looking at this cross sectional map of the WC responsiveness mechanisms.

###  How Seriously Is Feedback Taken by the Health System?

As noted, there seems to be a general lack of evaluation of the utilization and effectiveness of these mechanisms. For example, while having a functional health committee was a criteria for Ideal Clinic status, there appeared to be little clarity on what a ‘functional health committee’ was, or how this should be assessed. Generally, the utilization or effectiveness of the mechanisms to gather public feedback or illicit systemic response was unknown. This is not unique to this particular case or context. Loewenson reminds, “The simple assembly of stakeholder fora to elicit view or gather information does not constitute the form of participation in the governance of health systems that is increasingly being demanded.”^56^ This point resonates in SA and in LMICs more broadly, where participation is often “…largely spectator politics, where ordinary people have mostly become endorsees of pre-designed planning programs.”^57^ Considering the inequities in these systems, it is critical that there is deeper investigation into whose voices are being heard (whose feedback is gathered), and even more importantly, what response is generated – and what barriers prevent the intended loops of feedback and response.

Even more concerning: while there is overt and legislated support for the gathering of public feedback, and a legislated mandate that this should result in service- and systems-level *response*, in fact, the evidence gaps indicated above, and the absence of publicized routine monitoring across all these mechanisms, is the strongest indication that public input is still seen as ‘less important’ and something to be speedily ‘resolved away’ (as appears to be the case with complaints) – rather than an organized system channeling and collating valuable feedback into a ‘learning system.’

###  Barriers to Mechanism Functionality

Similar to the provincial case, at a national level in SA, it is acknowledged that public participation in the health system is still under-developed, and that the mechanisms intended to support this face massive implementation barriers, including a basic lack of information about their functioning.^15,58^ Common barriers found in this case, are echoed in the broader national and regional literature. For example, resource constraints are the most prominent factor: expressed in relation to the sustainability of committees, the functioning of the Health Ombud,^25^ and the lack of formalization of the CHWs.^39^ The global literature shows clearly that responsiveness mechanisms require financial support for implementation and sustainability – and furthermore, in SA, health expenditure directly correlates with evidenced health outcomes.^59^ A barrier that is not mentioned, but seems apparent, is that while the resourcing of the basic mechanism is sometimes addressed, usually in terms of its establishment, we found no mention of the resourcing of the service or systems response. This seems to be another potential barrier.

A related barrier was human resourcing – the resourcing of, or accountability lines indicated – with much of the policy documentation missing any mention of the actors that are supposed to be responsible for functioning of these mechanisms, or the response to feedback (Box 4).^26,60-62^

**Box 4. Actors at the Heart of Mechanisms^28,59-61^**

The WCDOH places emphasis on timely responses to complaints (20 minutes–2 hour turnaround time via telephonic/SMS/email hotline). This is a tight timeframe for a heavily-burdened and under-resourced health system and a complaints system that does not allocate dedicated human resources to resolution and response. This needs to be contextualized within the challenges SA health provider face:

- Medical equipment shortages
- Failing infrastructure
- Lack of funding
- Poor management and neglect
- Poor information management
- Staff shortages

Information on how staff manage the added work from feedback mechanisms within day to day roles is missing.

Abbreviations: SA, South Africa; WCDOH, Western Cape Department of Health.

Language was also reconfirmed as a barrier to responsiveness. For example, in a survey undertaken in a large, urban pediatric hospital in Cape Town, where 94% of medical interviews with the parents of patients were conducted in their second or third language, “parents cited language and cultural barriers, rather than structural and socioeconomic barriers, as the major barriers to their effective participation in the healthcare rendered to their children.”^63,64^ Another study at a WC district hospital found that language barriers hindered effective workings within the hospital and created misunderstandings between patients and staff, despite the fact that an official language policy is in place in the province.^65^ While the issues of language and service quality is being addressed in the literature – how language acts as a barrier to health system responsiveness, especially in multi-lingual LMIC settings, is underexplored.

###  Taking “Informal” Feedback Into Account

Taking a whole-systems view of mechanisms and responsiveness highlights that ‘informal’ (unlegislated) channels for feedback might be important,^14^ such as the media, protests or strike action illustrated earlier. A study on media coverage of maternal health in Bangladesh, Rwanda and SA, for example, found an association between the amount and type of media coverage and progress on the Millennium Development Goal 5.^66^ In the United States of America, there is evidence that social media is revolutionizing health-seeking behavior and practice.^67^ In SA there have only been a few studies examining the connection between media and health system,^48^ and the SA NHRD database shows no current studies focusing on media/social media, apart from a small study on the use of social media among nursing students in KwaZulu Natal.^68^ In a world where media and social media (the latter arguably governed by the public), is increasingly utilized, media as a channel for feedback and a potential lever for responsiveness needs to be explored further. Furthermore, the case reports of verbal and physical abuse experienced by health providers mirrors LMIC trends. For example, in 2003, Steinman found that 71% of health providers in public health facilities had faced violence in the workplace.^69^ Strikes, protest, and violence, can all be understood as a form of ‘feedback’ in which the public might be expressing dissatisfaction, perhaps not being able to give voice through other formal mechanisms, and are seeking some form of response – but this remains a largely unexplored area.^14^

###  Limitations

 This descriptive scoping review covered a broad terrain and sought to synthesize multiple forms of evidence. However, this approach also limits some of the conclusions that can be made. For example, while we have reviewed the available evidence, in several places, the lack of available data made it impossible to conclusively assess the ‘level’ of responsiveness in this particular system – not in a way that a future cross sectional assessment could useful provide a comparison. All that could be compared is the ‘configuration’ of responsiveness mechanism, which would be useful, but not explanatory. The evidence gathered here also highlights the need for further empirical research – in particular to understand local implementation practice in more detail.

## Conclusion

 This scoping review described the current ‘picture’ of mechanisms that are intended to support health system responsiveness in a particular local health system context. It found robust policies and guidelines in place for many mechanisms, but massive policy-implementation gaps, and many unanswered questions about functionality of these mechanisms, especially whether they support the development of a more responsive health system or not.

Generally, we found a lack of ‘whole-system’ perspective relating to whole-systems responsiveness. For example, mechanisms being assessed in isolation, different types of feedback being channeled in different directions, a general lack of routinized monitoring and ‘holes’ in feedback loops: between levels of the health system, between health systems, between mechanism types and missing various types of systems actors. It is impossible to check which parts of the public are being heard and who is being silenced, without looking across multiple mechanisms and routinely checking who is giving what feedback and whose feedback the system is receptive to, and responds to. It is also important to consider informal feedback via different channels alongside formally legislated and invited feedback via the mechanisms described above – and consider how decisions are made and policy developed with that in mind.^15^ It appears unlikely that any group or person in this provincial system has a full and adequate picture of all the feedback flowing through the system; of where the gaps and silenced voices exist; of what the trends are over time – to then be able to make systems-strengthening decisions. We did not find evidence of such an integrated system in the broader literature either.

This lack of whole-systems perspective was not only an observation of the actual mechanisms in this local system, but also the global and LMIC-focused research about such mechanisms and about health systems responsiveness seems to be similarly lacking in systems-thinking, or at least lacking enough macro ‘whole of systems’ perspective. We need much more extensive empirical and explanatory work to be conducted in specific systems – to check whether health systems are adequately responsive, to provide better explanations for some of the ideas suggested above – such as the idea that increased feedback through more mechanisms equals a more responsive health system. Most importantly, there needs to be a much greater focus on the other ‘end’ of the feedback loop – namely the systems response (or lack thereof). This was a massive and concerning gap in this case, and seems to be the same across other LMIC settings. We simply do not know how seriously public suggestions are being taken, or whether the health system is ‘learning’ from the feedback it receives and adapting accordingly. We still do not know whether public input is having any real effect on the health system.^14^ Lodenstein et al define health system responsiveness as, “…*the extent* to which a health provider or health policy-maker demonstrates *receptivity* to the ideas and concerns raised by citizens by *implementing changes* to the decision-making or management structure, culture, policies or practices.”^70^ This emphasis on the response is the next objective for health system actors and researchers alike.

## Acknowledgements

 This study originated as part of a University of Cape Town thesis – and we would like to thank the examiners involved in that work. This study is also linked to a broader study funded by the UK MRC ‘Strengthening health system responsiveness to community and citizen feedback in SA and Kenya’ (UKRI: MR/R013365/1) – and we would like to acknowledge the research and health systems partners involved in that broader study. Finally, to the Chaminuka Community Health System colleagues and editorial team, for the time spent thinking, and pulling this special edition together.

## Ethical issues

 This was a minimal risk study. Ethical clearance was still obtained from the University of Cape Town´s Human Research Ethics Committee (HREC reference 790/2019). No further ethical issues to report.

## Competing interests

 Authors declare that they have no competing interests.

## Authors’ contributions

 TS conducted the data collection, first round of analysis, and wrote the first draft. JO supervised the project, supported analysis, and wrote sections of the secondary drafts.

## Supplementary files


Supplementary file 1. PRISMA Checklist and Diagrams.

Supplementary file 2. Full List of South African National, Provincial and District Policies, Guidelines and Legislation Outlining Mechanisms for Receiving and Responding to Citizen Feedback.

Supplementary file 3. Contact Information for Complaints, Compliments and Suggestions in the Western Cape – A Comparison of 2015 and 2019.

Supplementary file 4. Table of Included Items From Scoping Review.
