# Supplementary material for: Mapping the Multiple Health System Responsiveness Mechanisms in One Local Health System: A Scoping Review of the Western Cape Provincial Health System of South Africa
Source: Int J Health Policy Manag. 2021 Aug 21;11(1):67–79. doi: 10.34172/ijhpm.2021.85 (PMC9278388; doi:10.34172/ijhpm.2021.85)
Supplement: Supplementary file 1 — PRISMA Checklist and Diagrams. [file ijhpm-11-67-s001.pdf]

**Article title:** Mapping the Multiple Health System Responsiveness Mechanisms in One Local Health System: A Scoping Review of the Western Cape Provincial Health System of South Africa

**Journal name:** International Journal of Health Policy and Management (IJHPM)

**Authors' information**: Tammy Sutherns*, Jill Olivier

Division of Health Policy and Systems, School of Public Health and Medicine, University of Cape Town, Cape Town, South Africa.

(*Corresponding author: tlsuth@gmail.com)

**Supplementary file 1.** PRISMA Checklist and Diagrams.

Adapted from: Moher D, Liberati A, Tetzlaff J, Altman DG, The PRISMA Group. Preferred Reporting Items for Systematic Reviews and Meta-Analyses: The PRISMA Statement. PLoS Med. 2009;6:1 -6. doi.org/10.1371/journal.pmed.1000097

| **Section/topic** | **#** | **Checklist item** | **Reported on page #** |
|---|---|---|---|
| **TITLE** | | | |
| Title | 1 | Propose a short take-home title. The title should explicitly state that the review included different types of evidence | 1 |
| **ABSTRACT** | | | |
| Structured summary | 2 | Provide a structured summary including, as applicable: background; objectives; data sources; study eligibility criteria, participants, and interventions; study appraisal and synthesis methods; results; limitations; conclusions and implications of key findings; systematic review registration number. | 1 |
| **INTRODUCTION** | | | |
| Rationale | 3 | Describe the rationale for the review in the context of what is already known. | 2 |
| Objectives | 4 | Formulate questions and/or objectives (qualitative, quantitative or both) being addressed by your review. | 3 |
| **METHODS** | | | |
| Protocol and registration | 5 | Indicate if a review protocol exists, if and where it can be accessed (e.g., Web address), and, if available, provide registration information including registration number. | 4 |
| Justification | 6 | Justify the use of a review of qualitative and quantitative evidence. | 4 |
| Eligibility criteria | 7 | Specify the inclusion and exclusion criteria and the rationale for supporting these criteria. | Supplementary files |
| Information sources | 8 | Describe all information sources (e.g., databases with dates of coverage, contact with study authors to identify additional studies) in the search and date last searched. | 4 |
| Search | 9 | Present full electronic search strategy for at least one database, including any limits used, such that it could be repeated.<br>Describe the process for removing duplicates | Supplementary files |
| Study selection | 10 | Describe the process for selecting studies (e.g. Screening based on titles and abstracts, and eligibility based on full-text, number of reviewers, software used) | Supplementary files |

| | | | |
|---|---|---|---|
| Data collection process | 11 | Describe the method of data extraction from included studies (e.g. number of reviewers involved, piloted forms, etc.).<br>List the data extracted | Supplementary files |
| Appraisal | 12 | Describe the process for appraising included studies (e.g., number of reviewers involved), and specifically for assessing the methodological quality or risk of bias of included qualitative, quantitative and mixed methods studies<br>Specify how results of this appraisal are used in the synthesis | Supplementary files |
| Synthesis | 13 | Describe the synthesis design used<br>Describe and justify the synthesis method (s) used | Supplementary files |

| | | | |
|---|---|---|---|
| **RESULTS** | | | |
| Study selection | 14 | Give numbers of studies screened, assessed for eligibility, and included in the review, with reasons for exclusions at each stage, ideally with a flow diagram. | Supplementary files |
| Study characteristics | 15 | For each study, present characteristics for which data were extracted (e.g., tables of characteristics included studies) and provide citations<br>Specify common information across all included studies | Supplementary files |
| Results of synthesis | 16 | Present results of synthesis | 5 |
| **DISCUSSION** | | | |
| Summary of evidence | 17 | Summarize an overall summary of results (take-home messages) from the qualitative and/or quantitative synthesis<br>State the main results for each main theme or category, and/or key process/outcome variable<br>Consider their relevance and importance for knowledge users (e.g., health care providers, managers, and decision/policy makers)<br>Take into account the methodological quality across studies (when applicable)<br>Describe insight gained from the integration of qualitative and quantitative evidence | 16 |
| Contribution | 18 | Describe the contribution of the review (compared to what is already known) with respect to: review methods, scientific knowledge, practice, program planning and evaluation, policy making or else. | 14 |
| Limitations | 19 | Discuss limitations at study and outcome level (e.g., risk of bias), and at review-level (e.g., incomplete retrieval of identified research, reporting bias). | 18 |
| Conclusions | 20 | Provide a general interpretation of the results in the context of other evidence, and implications for future research. | 19 |
| **REFERENCES** | | | |
| References | 21 | List all of the references cited in the text | 19 |
| **APPENDICES** | | | |

| | | |
|---|---|---|
| Flow diagram: Secondary literature: Peer-reviewed articles | 2<br>2 | 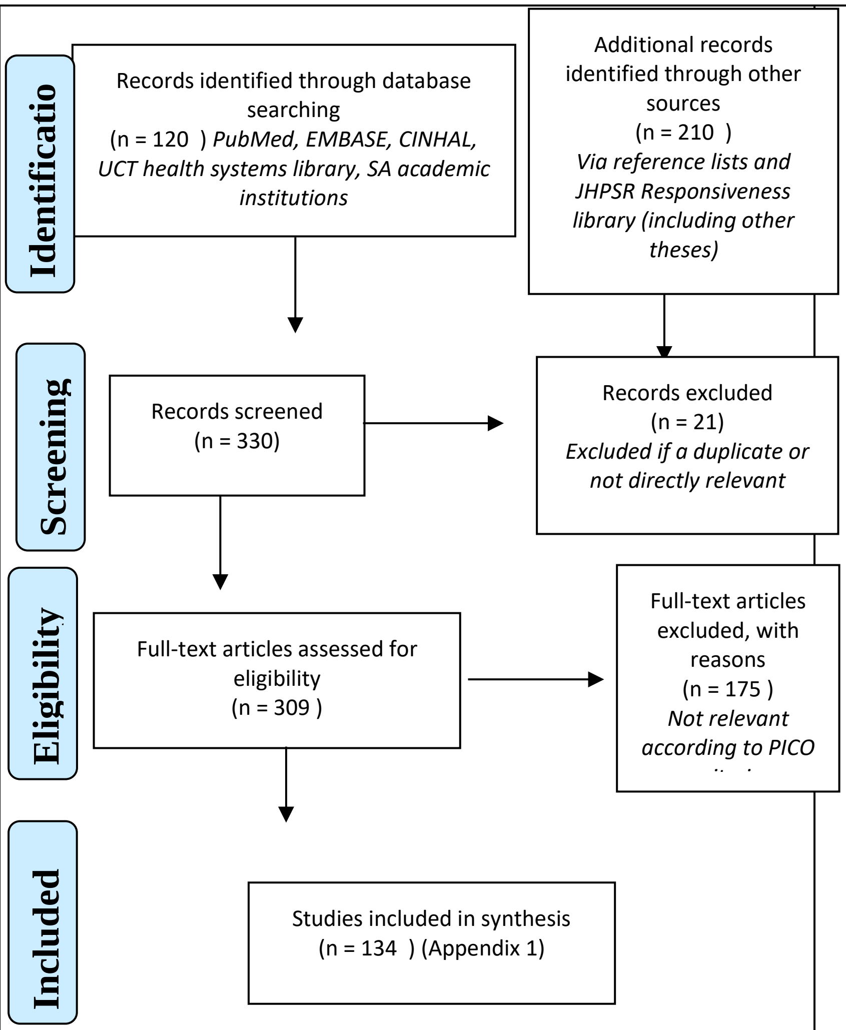 |

| | | | |
|---|---|---|---|
| Flow diagram: Secondary literature: Institutional reports | 23 | 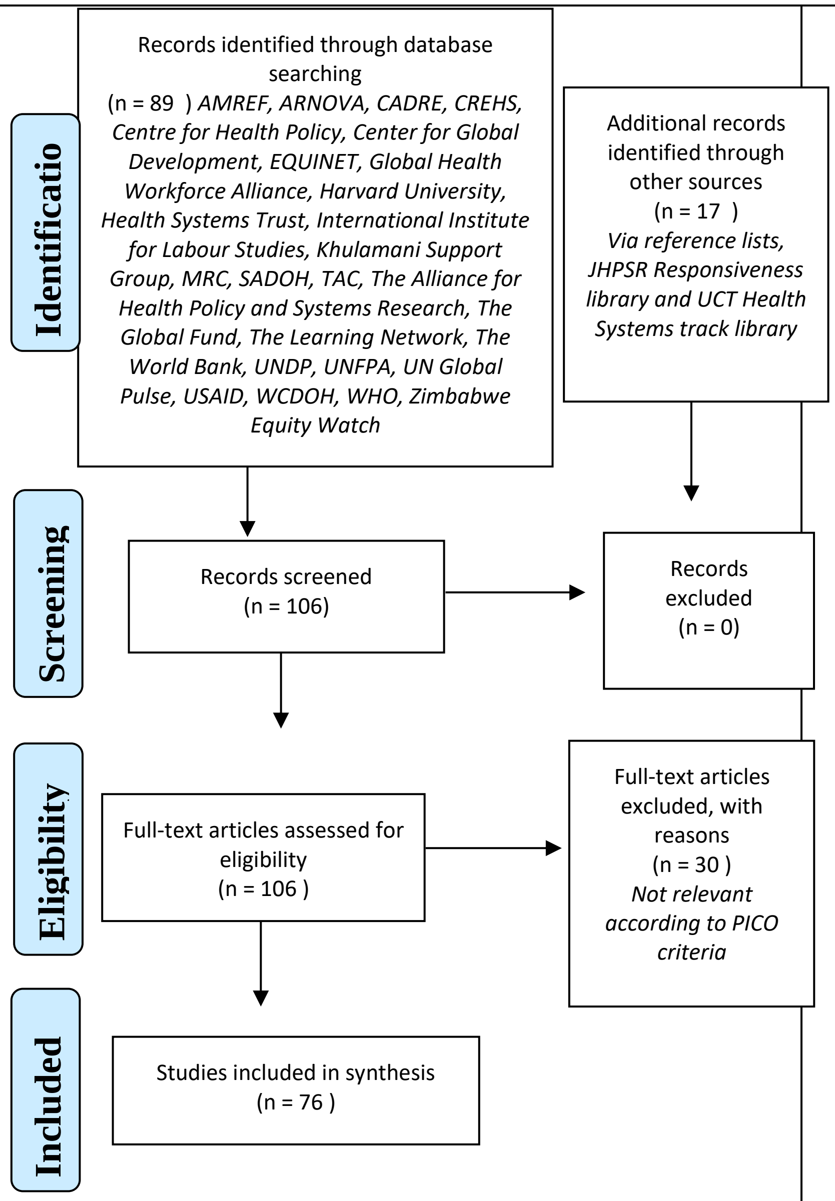 | |

| | | | |
|---|---|---|---|
| Flow diagram: SA/WC policy documents | 24 | 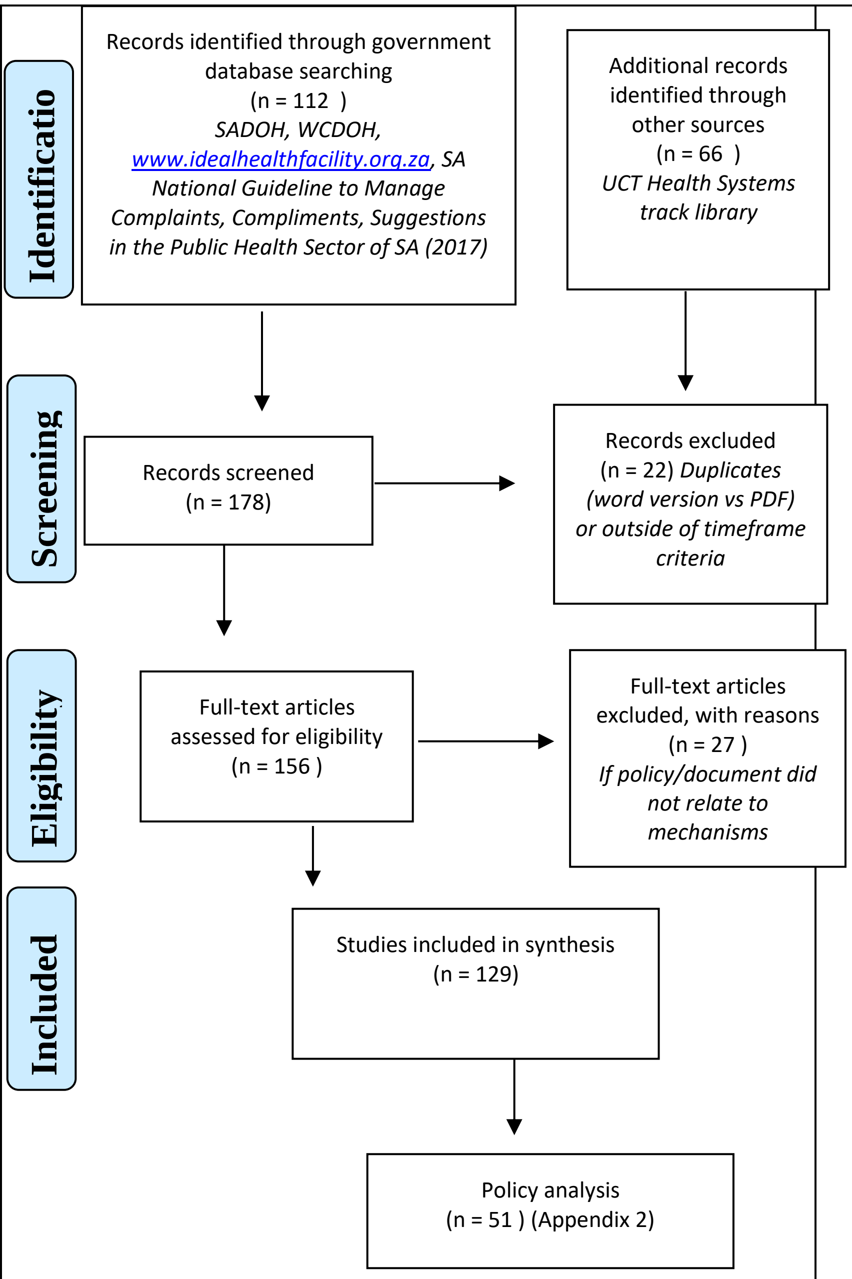 | |

| | | | |
|---|---|---|---|
| Flow diagram: Survey data | 26 | 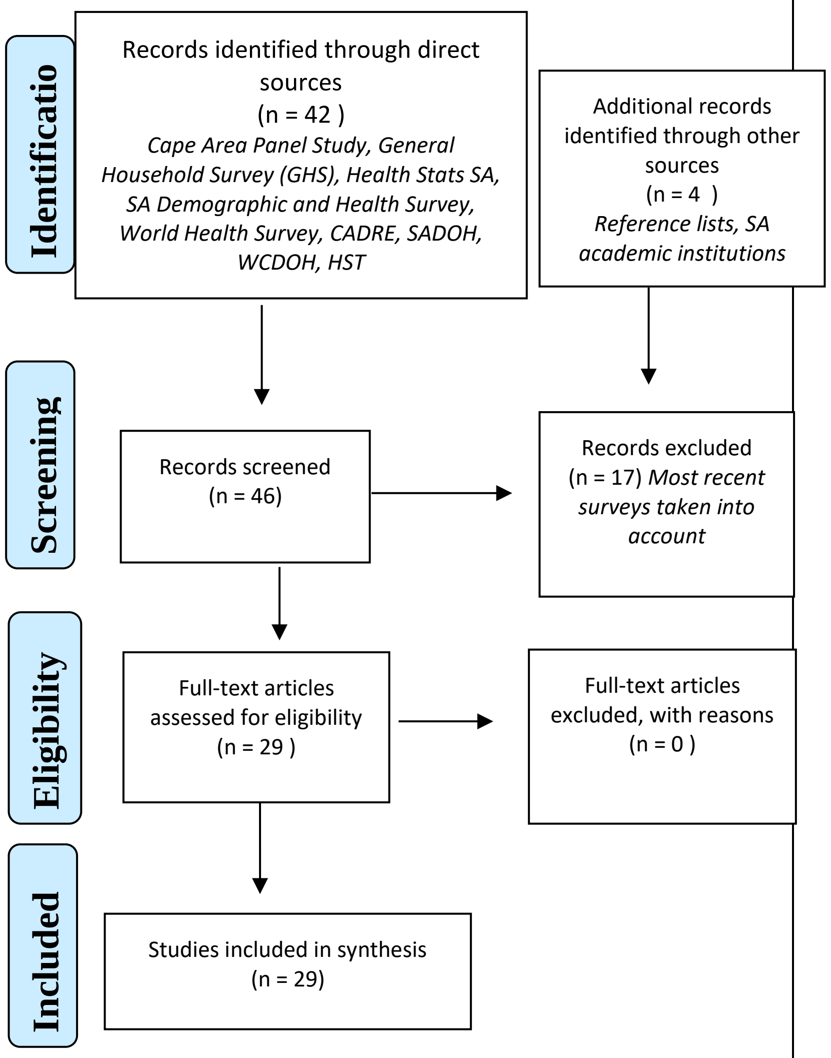 | |

<table>
<tr><td>Flow diagram: Media reports</td><td>27</td><td>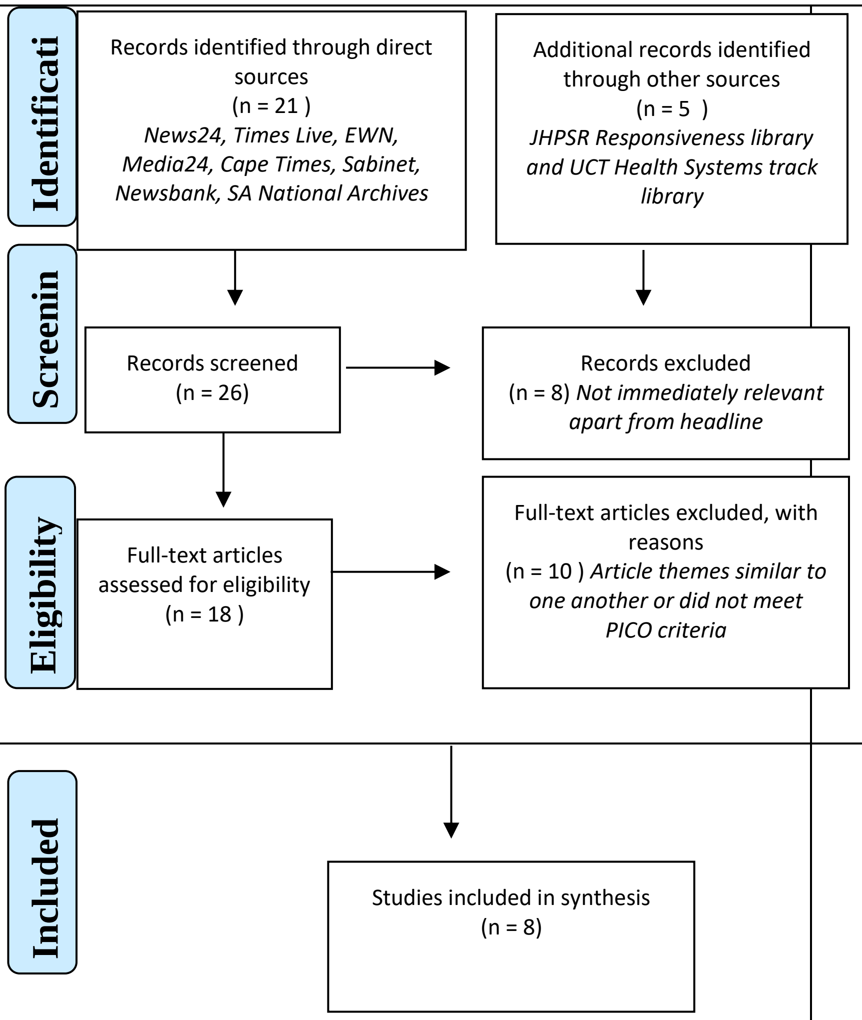
</td><td></td></tr>
<tr><td>Table of inclusion criteria</td><td>28</td><td>Table 1: PICO framework for inclusion criteria
<table>
<tr><td>P</td><td>Stakeholders in LMIC health systems, notably in South Africa</td></tr>
<tr><td>I</td><td>Any mechanism that facilitates citizen feedback in the health system. Due to the variety of mechanisms that do exist, they will also be searched for individually, including patient / client satisfaction surveys, health surveys, suggestion boxes, health committees, satisfaction interviews, media reports, protests, scorecards</td></tr>
<tr><td>C</td><td>Any or no comparator between health system responsiveness mechanisms will be eligible for inclusion</td></tr>
<tr><td>O</td><td>These include feedback being recorded or tracked as well as responded to or resolved</td></tr>
<tr><td>Extra criteria</td><td>• English materials<br>• Published from 2000 – 2019 (unless reference or trace-searching resulted in earlier relevant materials)</td></tr>
</table></td><td></td></tr>
</table>

PICO framework source: Schardt C, Adams MB, Owens T, Keitz S, Fontelo, P. Utilization of the PICO framework to improve searching PubMed for clinical questions. *BMC Med Inform Decis Mak.* 2007;7:1-6. doi: 10.1186/1472-6947-7-16
