# Supplementary material for: Mapping the Multiple Health System Responsiveness Mechanisms in One Local Health System: A Scoping Review of the Western Cape Provincial Health System of South Africa
Source: Int J Health Policy Manag. 2021 Aug 21;11(1):67–79. doi: 10.34172/ijhpm.2021.85 (PMC9278388; doi:10.34172/ijhpm.2021.85)
Supplement: Supplementary file 2 — Full List of South African National, Provincial and District Policies, Guidelines and Legislation Outlining Mechanisms for Receiving and Responding to Citizen Feedback. [file ijhpm-11-67-s002.pdf]

**Article title:** Mapping the Multiple Health System Responsiveness Mechanisms in One Local Health System: A Scoping Review of the Western Cape Provincial Health System of South Africa

**Journal name:** International Journal of Health Policy and Management (IJHPM)

**Authors' information**: Tammy Sutherns*, Jill Olivier

Division of Health Policy and Systems, School of Public Health and Medicine, University of Cape Town, Cape Town, South Africa.

(*Corresponding author: tlsuth@gmail.com)

**Supplementary file 2.** Full List of South African National, Provincial and District Policies, Guidelines and Legislation Outlining Mechanisms for Receiving and Responding to Citizen Feedback

| **Policy/ evidence** | **Mechanisms** | **Responsiveness** | **Level** |
|---|---|---|---|
| SADOH White Paper[1] | Comprehensive Primary Health Care<br>Decentralization (District Health System)<br>NGOs<br>Committees<br>Community-based information systems<br>Health summits | Transformation of national health system, community participation in planning/provision of health care on each level of government. | National, provincial, district |
| SA´s Patients' Rights Charter[2] | Information material | | National, provincial, district |
| WC Health Facility Boards Act, 7 of 2001[3] | Health Facility Boards | Ensure accountability of health facility management to community, responsiveness to needs of patients/their families. | Provincial |
| WC Government Health Annual Report 2002 – 2003[4] | Monitoring client/patient complaints and compliments | To identify the strengths/weaknesses of service delivery.<br>Creation of HFBs achieved throughout the province, promotes representation, accessibility, openness and transparency.<br>Findings of annual survey analysed, | Provincial |

| | | | |
|---|---|---|---|
| | | addressed with quality improvement plans formulated. | |
| SA National Health Act No. 61 of 2003[5] | Decentralisation<br>District health councils<br>Hospital Boards<br>Health Committees<br>Health Professional´s Council<br>Ombud<br>Office of Health Standards Compliance (OHSC) | Governance between national/provincial/local spheres, PHC approach/district health system model, decentralisation. Minister must appoint HFBs for each central hospital or group of hospitals, including 3 representatives of the communities served by the hospitals. | National, provincial, district |
| Having your say: A handbook for Ward Committees[6] | Ward committees | Representative structure of community/citizens, need to inform the municipality about aspirations, potentials and problems of the people. | Provincial, District |
| WC Government Health Annual Report 2004 – 2005[7] | CSS | Report evaluates percentage of facilities that conducted external client satisfaction survey, published results, developed action plans for improvement. | Provincial |
| WC Government Health Annual Report 2006- 2007[8] | Annual CSS – every 12 months | Negative feedback reported on, including client safety, waiting times, public transport, along with strategies for addressing these. | Provincial |
| WC Health Facility Boards – Manual 2007[9] | | Highlights how Board is accountable to community, patients, their families. | Provincial |
| WC Government Health Annual Report 2007 – 2008[10] | Annual PSS – every 12 months<br>Staff satisfaction survey | Analysis of patient satisfaction survey, interventions planned for responses & for results of staff survey. | Provincial |
| WC Government Health Annual Report 2008 – 2009[11] | Annual PSS – every 12 months | Performance measure indicator looks at how many complaints | Provincial |

| | | | |
|---|---|---|---|
| | | resolved within 25 days. Findings of annual survey analysed, addressed with quality improvement plans formulated. | |
| WC Government Health Annual Report 2009 – 2010[12] | Annual PSS – every 12 months<br>Complaints mechanism: Departmental complaints procedure and suggestion box | All complaints should be responded to within one month Suggestion box serves a mechanism where suggestions are recorded and discussed weekly with action plans developed by Facility Management. Findings of annual survey analysed, addressed with quality improvement plans formulated. | Provincial |
| Draft Regulations Relating to the Functioning of the District Health Councils in terms of the Western Cape District Health Councils Act, 2010 | | | Provincial |
| Western Cape District Health Councils Act, 5 of 2010 | | | Provincial |
| "Towards Quality Care for Patients" National Core Standards for Health Establishments in SA[13] | District Health Information System, audit tool & audit team<br>Hospital Board<br>Clinic Committee<br>Community Health Forums<br>PSS<br>Patient complaints<br>Feedback & forums | Standards for quality health services, measures compliance on six standards. | National |
| 2020: The future of health care in the WC[14] | | CHWs as direct link between family, community and health service. | Provincial |
| WC Government Health Annual Report 2010 – 2011[15] | Annual PSS – every 12 months<br>Complaints committee<br>Independent Complaints Commission | MEC/Head of Department can refer complaints when internal processes for dealing with complaints have not addressed the issues to satisfaction of complainant. Findings of annual survey | Provincial |

| | | | |
|---|---|---|---|
| | | analysed, addressed with quality improvement plans formulated. | |
| WC Health Facility Boards Amendment Act, 7 of 2012 | | | Provincial |
| Public Service Regulations, 2001[16] | Citizens' Complaints and Compliments Framework, March 2013 | Stipulates exec authority shall establish/sustain service delivery programme for his/her dept– one key element is system or mechanism for (managing) complaints & compliments. | National |
| 20 Year Review: WC[17] | Ward committees<br>SMS-hotline<br>Complaints & compliments process<br>Client satisfaction surveys | Reviews transformation since 1994 against the Batho Pele Principles. | Provincial |
| WC District Health Councils Amendment Act, 2013[18] | District Health Councils | Amend 2010 Act to include members of health subdistricts in a district health councils. | District |
| WC Government Health Annual Report 2012 – 2013[19] | Annual client satisfaction surveys (CSS)– every 12 months<br>Complaints SMS and telephone hotline<br>Draft Western Cape Independent Health Complaints Committee Bill<br>Independent Complaints Committee | Hotline piloted, complaints logged/tracked. A system for the referral of complaints.<br>Findings of annual survey analysed, addressed with quality improvement plans formulated. | Provincial |
| WC Independent Health Complaints Committee Regulations, 2014 | | | Provincial |
| Healthcare 2030: The Road to Wellness[20] | SOP<br>Public engagement<br>Social protest action<br>Community communication mechanisms<br>PSS<br>Mechanisms for written, oral or telephonic complaints & compliments<br>Rapid surveys<br>Health hotline<br>District councils<br>Facility boards | Department´s values include responsiveness.<br>District Councils enable effective communication within the community.<br>Facility boards enable effective communication within the community. | Provincial |

| | | | |
|---|---|---|---|
| | Clinic committees | | |
| WC Government Health Annual Report 2013 – 2014[21] | Annual CSS – every 12 months<br>Complaints hotline<br>Independent Health Complaints Committee<br>NGO/NPOs<br>Assessments against National Core Standards | Complaints referred to Committee for consideration. Report looks at number of compliments/complaints received. Information/complaints process displayed on notice boards at clinic. NGO utilised. Reports on patient satisfaction. | Provincial |
| WC Independent Health Complaints Committee Act, 2 of 2014[22] | Independent Health Complaints Committee | Establishment of Independent Health Complaints Committee. | Provincial |
| WC Government Health Annual Report 2014 – 2015[23] | Annual CSS – every 12 months<br>Western Cape Independent Complaints Committee Act<br>Designated complaints champion/officer | Complaints champion/officer ensures compliance with the 25 day resolution date. | Provincial |
| Regulations Governing the Financial Prescripts in terms of Western Cape Health Facility Boards and Committees Act, 2016 | | | District |
| Manual in terms of section 14 of the Promotion of Access to Information Act, 2000[24] | Citizen rights | Right of access to information. | Provincial |
| WC Government Health Annual Report 2015 – 2016[25] | Annual CSS – every 12 months | Findings of annual survey analysed, addressed with quality improvement plans formulated. | Provincial |
| WC Health Facility Boards and Committees Act, 2016[26] | Health Facility Boards<br>Committees | Committee duties. Health Facility Board duties. | Provincial legislation |
| Regulations relating to the Criteria and Process for the Clustering of Primary Health Care Facilities in terms of the Western Cape Health Facility Boards and Committees Act, 2017 | | | District |
| Regulations Governing the Procedures for the Nomination of Members for Appointment to Health Facility Boards in terms of the Western Cape Health Facility Boards and Committees Act, 2017 | | | Provincial |
| SADOH Annual Report 2016/2017<br>*Annual Report 2016/2017*[27] | Complaints/compliments procedures<br>*MomConnect* programme | Benchmarks SADOH progress, including | National |

| | | | |
|---|---|---|---|
| | National Toll-Free Complaints Call Centre | improvement in responsiveness. | |
| National Guideline to Manage Complaints, Compliments and Suggestions in the Public Health Sector of South Africa[28] | Complaint, complement, suggestion procedure<br>Public Protector<br>Consumer Commission<br>Human Rights Commission<br>Legal System<br>Ombud in the OHSC<br>Professional Councils/Boards<br>Feedback forms<br>Complaint/compliment/suggestion boxes<br>Posters & pamphlets detailing feedback process<br>Complaint, Compliment and Suggestion Committee (CCSC) | Right to complain about healthcare received, guidelines/standards monitor whether health facilities adhere. | National |
| WC Government Health Annual Report 2016- 2017[29] | Provincial Health Council, District Health Councils, Hospital Boards<br>WC Health Facility Boards and Clinic Committees Act Regulations<br>Community Care Workers<br>Health Committee<br>Patient Satisfaction Survey<br>Complaint Resolution Rate<br>National Core Standards | Consultations/meetings with Health Committee, including Community Questions Answers Session. | District |
| SADOH Annual Report 2017/2018[30] | *MomConnect* programme<br>OHSC<br>National Health Council (NHC)<br>Complaints/compliments procedure<br>National Survey: to measure Patient Experience of Care | Management of client complaints, suggestions and compliments, annual health facility surveys of patients´ experience of care, health facility monitoring and reporting of Patient Safety Incidents. | National |
| Annual Inspection Report 2016/2017 and Annual Inspection Report 2018/2019[31,32] | OHSC<br>Annual Inspection Report<br>Complaint process<br>Complaints management and Ombud | Monitors delivery of safe, quality care in compliance with the National Core Standards.<br>OHSC protects health/safety of users by investigating complaints. Ombud reviews annual inspections. | National |
| Cape Metro District 2018: District Health Plan 2018/2019 to 2020/21[33] | Patient complaints<br>National Core Standards | Highlights priorities against National Core Standards. Monitors | District |

| | | | |
|---|---|---|---|
| | Complaint and Compliment Register<br>District Health Committee<br>Clinic Committee and Facility Boards<br>NPOs | complaints resolved within 25 days. | |
| Cape Winelands District 2018: District Health Plan 2018/2019 to 2020/21[34] | Patient complaints<br>National Core Standards<br>Complaint and Compliment Register<br>District Health Committee<br>Clinic Committee and Facility Boards | Highlights priorities against National Core Standards. Monitors complaints resolved within 25 days. | District |
| Central Karoo District 2018: District Health Plan 2018/2019 to 2020/21[35] | Patient complaints<br>National Core Standards<br>Complaint and Compliment Register<br>District Health Committee<br>Clinic Committee and Facility Boards | Promotes principles of Community Orientated Primary Care. Highlights priorities against National Core Standards. Monitors complaints resolved within 25 days. | District |
| Eden District 2018: District Health Plan 2018/2019 to 2020/21[36] | Patient complaints<br>National Core Standards<br>Complaint and Compliment Register<br>Clinic Committee and Facility Boards | Promotes principles of Community Orientated Primary Care. Highlights priorities against National Core Standards. Monitors complaints resolved within 25 days. | District |
| Overberg District 2018: District Health Plan 2018/2019 to 2020/21[37] | Patient complaints<br>National Core Standards<br>Complaint and Compliment Register<br>Clinic Committee and Facility Boards | Highlights priorities against National Core Standards. Monitors complaints resolved within 25 days. | District |
| West Coast District 2018: District Health Plan 2018/2019 to 2020/21[38] | Patient complaints<br>National Core Standards<br>Complaint and Compliment Register<br>Clinic Committee and Facility Boards | Highlights priorities against National Core Standards. Monitors complaints resolved within 25 days. | District |
| WBPHCOT Policy Framework and strategy[39] | Ward-based Primary Health Care Outreach Teams (WBPHCOT) | WBPHCOT intended to support delivery of PHC in South Africa. | National |
| WC Government Health Annual Report 2017 – 2018[40] | Community Care Workers<br>National Core Standards<br>Complaint system & Resolution Rate<br>Patient Satisfaction Survey<br>Waiting Time Survey Report | Intention to have committees up and running by new financial year. The WC HFBs and Committee Act was | District |

| | | | |
|---|---|---|---|
| | Health Facility Boards<br>Clinic Committees | promulgated in 2016 and regulations gazetted on 7 December 2017. Complaint resolution rate measured. Monthly Data Review and Facility Manager meetings. National Guideline implemented. | |
| National Health Insurance Bill 2019[41] | Complaints<br>Stakeholder Advisory Committee | To achieve universal access to quality health care services. | National |
| *What is MomConnect?*[42] | MomConnect | Interactive mechanism to provide feedback on services. | National |
| WC Government Health Annual Report 2018 – 2019[43] | | With WC Health Facility Boards and Committee Act promulgated in 2016, establishment of clinic committees commenced in January 2018 and implementation work in progress. | District |
| Office of the Health Ombud Annual Report 2018/2019[44] | OHSC<br>Health Ombud | Reports on progress of the OHO to protect and promote the health/safety of users of healthcare in SA by investigating & reporting on complaints. | National |
| Office of the Health Ombud website http://healthombud.org.za/[45] | Online complaints submission portal<br>Complaints Call Centre | Online platform for citizens to learn more about the Health Ombud, lay complaints, access reports, publications, other resources. | National |

**References:**


1. South African Department of Health. *White Paper for the Transformation of the Health System in South Africa*. South Africa: South African Department of Health; 1997.
2. South African Department of Health. *Patient Rights Charter*. South Africa: South African Department of Health; 1999.
3. Western Cape Department of Health. *Western Cape Health Facility Boards Act, 7 of 2001*. South Africa: Western Cape Department of Health; 2001.

4. Western Cape Department of Health. *Western Cape Government Health Annual Report 2002 - 2003*. South Africa: Western Cape Department of Health; 2003.
5. South African Department of Health. *National Health Act 61 of 2003*. Pretoria: National Government of South Africa; 2004.
6. South African Department of Health. *Having Your Say: A Handbook for Ward Committees*. South Africa: South African Department of Health; 2005.
7. Western Cape Department of Health. *Western Cape Government Health Annual Report 2004 - 2005*. South Africa: Western Cape Department of Health; 2005.
8. Western Cape Department of Health. *Western Cape Government Health Annual Report 2006 – 2007*. South Africa: Western Cape Department of Health; 2007.
9. Western Cape Department of Health. *Western Cape Health Facility Boards - Manual 2007*. South Africa: Western Cape Department of Health; 2007.
10. Western Cape Department of Health. *Western Cape Government Health Annual Report 2007 – 2008*. South Africa: Western Cape Department of Health; 2008.
11. Western Cape Department of Health. *Western Cape Government Health Annual Report 2008 – 2009*. South Africa: Western Cape Department of Health; 2009.
12. Western Cape Department of Health. *Western Cape Government Health Annual Report 2009 – 2010*. South Africa: Western Cape Department of Health; 2010.
13. South African Department of Health. *Towards Quality Care for Patients: National Core Standards for Health Establishments in South Africa*. South Africa: South African Department of Health; 2011.
14. Western Cape Department of Health. *2020: The Future of Health Care in the Western Cape*. South Africa: Western Cape Department of Health; 2011.
15. Western Cape Department of Health. *Western Cape Government Health Annual Report 2010 – 2011*. South Africa: Western Cape Department of Health; 2011.
16. South African National Department of Public Service and Administration. *Public Service Regulations, 2001*. South Africa: South African National Department of Public Service and Administration; 2013.
17. Western Cape Department of Health. *20 Year Review: Western Cape*. South Africa: Western Cape Department of Health; 2013.
18. Western Cape Department of Health. *Western Cape District Health Councils Amendment Act, 2013*. South Africa: Western Cape Department of Health; 2013.
19. Western Cape Department of Health. *Western Cape Government Health Annual Report 2012 – 2013*. South Africa: Western Cape Department of Health; 2013.
20. Western Cape Department of Health. *Healthcare 2030: The Road to Wellness*. South Africa: Western Cape Department of Health; 2014.
21. Western Cape Department of Health. *Western Cape Government Health Annual Report 2013 - 2014*. South Africa: Western Cape Department of Health; 2014.
22. Western Cape Department of Health. *WC Independent Health Complaints Committee Act, 2 of 2014*. South Africa: Western Cape Department of Health; 2014.
23. Western Cape Department of Health. *WC Government Health Annual Report 2014 – 2015*. South Africa: Western Cape Department of Health; 2015.
24. Western Cape Department of Health. *Manual in Terms of Section 14 of the Promotion of Access to Information Act, 2000*. South Africa: Western Cape Department of Health; 2016.
25. Western Cape Department of Health. *Western Cape Government Health Annual Report 2015 – 2016*. South Africa: Western Cape Department of Health; 2016.
26. Western Cape Department of Health. *Western Cape Health Facility Boards and Committees Act*. South Africa: Western Cape Department of Health; 2016.
27. South African Department of Health. *Annual Report 2016/2017*. South Africa: South African Department of Health; 2017.
28. South African Department of Health. *National Guideline to Manage Complaints, Compliments and Suggestions in the Public Health Sector of South Africa*. South Africa: South African Department of Health; 2017.

29. Western Cape Department of Health. *Western Cape Government Health Annual Report 2016 – 2017*. South Africa: Western Cape Department of Health; 2017.
30. South African Department of Health. *Annual Report 2017/2018*. South Africa: South African Department of Health; 2018.
31. Office of Health Standards Compliance. *Annual Inspection Report 2016/2017*. South Africa: OHSC; 2017.
32. Office of Health Standards Compliance. *Annual Inspection Report 2017/2018.* South Africa: OHSC; 2017.
33. Western Cape Department of Health. *Cape Metro District 2018: District Health Plan 2018/2019 to 2020/2021*. South Africa: Western Cape Department of Health; 2018.
34. Western Cape Department of Health. *Cape Winelands District 2018: District Health Plan 2018/2019 to 2020/2021*. South Africa: Western Cape Department of Health; 2018.
35. Western Cape Department of Health. *Central Karoo District 2018: District Health Plan 2018/2019 to 2020/2021*. South Africa: Western Cape Department of Health; 2018.
36. Western Cape Department of Health. *Eden District 2018: District Health Plan 2018/2019 to 2020/2021*. South Africa: Western Cape Department of Health; 2018.
37. Western Cape Government. *Overberg District 2018: District Health Plan 2018/2019 to 2020/2021*. South Africa: Western Cape Department of Health; 2018.
38. Western Cape Department of Health. *West Coast District 2018: District Health Plan 2018/2019 to 2020/2021*. South Africa: Western Cape Department of Health; 2018.
39. South African Department of Health. *WBPHCOT Policy Framework and Strategy*. South Africa: South African Department of Health; 2018.
40. Western Cape Department of Health. *Western Cape Government Health Annual Report 2017 - 2018*. South Africa: Western Cape Department of Health; 2018.
41. South African Department of Health. *National Health Insurance Bill 2019*. South Africa: South African Department of Health; 2019.
42. South African Department of Health. What is MomConnect? South Africa Department of health. Published 2019. http://www.health.gov.za/index.php/mom-connect. Accessed December 9, 2019.
43. Western Cape Department of Health. *Western Cape Government Health Annual Report 2018 - 2019*. South Africa: Western Cape Department of Health; 2019.
44. Office of the Health Ombud. *Office of the Health Ombud Annual Report 2018/2019*. South Africa: OHO
45. Office of the Health Ombud. Office of the Health Ombud. http://healthombud.org.za. Published 2020. Accessed December 12, 2019.
