# Supplementary material for: Mapping the Multiple Health System Responsiveness Mechanisms in One Local Health System: A Scoping Review of the Western Cape Provincial Health System of South Africa
Source: Int J Health Policy Manag. 2021 Aug 21;11(1):67–79. doi: 10.34172/ijhpm.2021.85 (PMC9278388; doi:10.34172/ijhpm.2021.85)
Supplement: Supplementary file 3 — Contact Information for Complaints, Compliments and Suggestions in the Western Cape – A Comparison of 2015 and 2019. [file ijhpm-11-67-s003.pdf]

Article title: Mapping the Multiple Health System Responsiveness Mechanisms in One Local Health System: A Scoping Review of the Western Cape Provincial Health System of South Africa

Journal name: International Journal of Health Policy and Management (IJHPM)

Authors' information: Tammy Sutherns*, Jill Olivier

Division of Health Policy and Systems, School of Public Health and Medicine, University of Cape Town, Cape Town, South Africa.

(*Corresponding author: tlsuth@gmail.com)

Supplementary file 3. Contact Information for Complaints, Compliments and Suggestions in the Western Cape – A Comparison of 2015 and 2019.

Source: WCDOH, Western Cape Government Health Annual Report 2018 – 2019.

**2015:**<br>(WCDOH, 2015)	**2019:**<br>(WCDOH, 2019a)
**For more information or assistance of any kind, please contact the:**<br>Western Cape Government Contact Centre:<br>• **Call:** 0860 142 142<br>- Monday to Sunday 07:00 - 19:00<br>- cost of a local telephone call from anywhere in South Africa<br>• **Fax:** 021 483 7216<br>• **SMS:** Help to 31022<br>• **Please Call Me:** 079 769 1207<br>• **Email:** service@westerncape.gov.za<br>Department of Health<br>• **Tel:** 021 483 3245<br>• **Fax:** 021 483 6169	**Contact Centre**<br>Queries or complaints about Western Cape Government services can be directed to the Western Cape Government Contact Centre. You can contact us through the channel that suits you such as social media, the walk-in centre or the call centre.<br>You can also email us, or submit your enquiry or complaint with our contact centre form. The contact centre will help direct your complaint or enquiry and make contact with the relevant agencies. Please note that we will try to deal with your matter as fast as possible, but turn-around times may vary depending on the complexity of your complaint or enquiry. You can visit our Walk-in Centre at 9 Wale Street, Mondays to Fridays, between 7:30am and 4pm.<br>**Call:** 0860 142 142<br>**Fax:** 021 483 7216<br>**SMS:** Help to 31022<br>**Please Call Me:** 079 769 1207<br>**Email:** service@westerncape.gov.za<br>Facebook: www.facebook.com/WesternCapeGovernment<br>Tweet us: twitter.com/WesternCapeGov<br>Or, alternatively, you can also complete an online form.<br>**Department of Health Complaints**<br>The Western Cape Government Department of Health offers a wide range of healthcare facilities across the province. Should you feel that they are not delivering services of sufficient standard, you are welcome to contact them via their patient complaints line.<br>• **SMS** the word "Help" – followed by your name, the nature of your complaint, facility and, if applicable, the name of a staff member to 31022.<br>• **Call:** 0860 142 142 and press "1".<br>• **Please Call Me:** 079 769 1207<br>• Send an **email** to service@westerncape.gov.za
