# Supplementary material for: Mapping the Multiple Health System Responsiveness Mechanisms in One Local Health System: A Scoping Review of the Western Cape Provincial Health System of South Africa
Source: Int J Health Policy Manag. 2021 Aug 21;11(1):67–79. doi: 10.34172/ijhpm.2021.85 (PMC9278388; doi:10.34172/ijhpm.2021.85)
Supplement: Supplementary file 4 — Table of Included Items From Scoping Review. [file ijhpm-11-67-s004.pdf]

**Article title:** Mapping the Multiple Health System Responsiveness Mechanisms in One Local Health System: A Scoping Review of the Western Cape Provincial Health System of South Africa

**Journal name:** International Journal of Health Policy and Management (IJHPM)

**Authors' information**: Tammy Sutherns*, Jill Olivier

Division of Health Policy and Systems, School of Public Health and Medicine, University of Cape Town, Cape Town, South Africa.

(*Corresponding author: tlsuth@gmail.com)

**Supplementary file 4.** Table of Included Items From Scoping Review.

| MECH | COUNTRY | OVERVIEW OF STUDY FOCUS AND FINDINGS |
|---|---|---|
| **Decentralisation (>10 studies)** | India | Village Health Sanitation and Nutrition Committees, participatory forums, intended to decentralise planning/action to improve community health, sanitation and nutrition. Lacking education, mobilisation and monitoring.[1] |
| | Mali | Gov of Mali´s decentralization of local health centre management to local institutions through delegation to community health association and the devolution of decisions to local govs. Key is responsiveness to local needs, downward accountability and health provider retention.[2] |
| | Kenya | Gov of Kenya´s 1994 Health Policy Framework, including decentralisation to the district level. There is not enough emphasis on process, health sector reforms unsustainable.[3] |
| | Nicaragua | Structural adjustments have accompanied health service decentralization, leading to a lack of equity and accountability. Deeper analysis of political and economic factors needed.[4] |
| | Pakistan | Study of decentralisation (authority, institutional capacities and accountability to local authorities), showing it occurs differently depending on local context.[5] |
| | Tanzania | Decentralisation of expanded programme on immunization (EPI). Shows community support depends on health provider availability and awareness of target population.[6] |
| | | Researchers investigate the decentralisation and control of tropical diseases, showing that devolution occurs more in theory than in practice.[7] |
| | LMICs | Factors influencing health provider accountability (oversight mechanisms, revenue sources and competition in the health sector). Findings show evidence is thin, official community participation mechanisms in context of health service decentralisation can improve responsiveness.[8] |
| | | Factors that influence how accountability mechanisms function and relationships within the district health system, importance of organisational culture. [9] |
| | | Links between governance mechanisms and health outcomes. Health system decentralisation is one key governance mechanisms that enables responsiveness to local needs and values.[10] |
| | | Challenges in health system strengthening interventions, applying a model of health governance, including principal-agent linkages. [11] |
| **Case review/audit (<5 studies)** | Côte d'Ivoire | Frequency of severe obstetric illness, intervals between admission or decision and life-saving surgery, factors contributing to delays – reported in case reviews in two hospitals.[12] |
| | LMICs | Theory-driven review of collective citizen engagement/advocacy cases, insight into perspectives, reasoning, agency, abilities of health providers to respond to citizens. Must evaluate intermediate effects (attitudinal/behavioural changes or social accountability initiatives). [13] |
| **Community Health Insurance (CHI) (<5 studies)** | India | Assesses patient satisfaction after hospitalisation for insured and uninsured patients. In reality, health insurance does not always lead to higher satisfaction. [14] |
| | Ghana | Assesses Ghana´s NHIS, challenges include sustainability, questions around equity, structure and accountability.[15] |
| | LMICs | Describes origins, formats and evolution of CHI in Africa, Asia & Latin America, including strengths & weaknesses. [16] |
| **Community Health Workers (CHWs) (>10 studies)** | Bangladesh | Examines how poor populations can access trusted knowledge and services in pluralistic health systems and role of CHWs, based on past successes and failures. Suggests four potential models of community-based health agents.[17] |
| | | Assesses feasibility and constraints of community-based management of acute malnutrition (CMAM), recommends it for MAM and SAM.[18] |

| | | |
|---|---|---|
| | Brazil | Assesses feasibility and effectiveness of CHW programmes through a desktop review.[19] |
| | Cambodia | Assesses if investment into Community Systems Strengthening has improved effectiveness, efficiency, results of HIV, TB, malaria programs.[20] |
| | Ethiopia | Community Health Systems Strengthening (CHSS) model utilises formal/informal networks in communities to address gaps in services. Can support and legitimise CTC (close-to-community) providers and create sustainable community-based programmes.[21] |
| | India | Explores perceptions/experiences of ASHA scheme (Accredited Social Health Activists) – a cadre of India´s CHW programme. Finds scheme is beneficial but faces challenges.[22] |
| | South Africa | Explores history of CHWs to inform policy-making frameworks for CHWs going forward. [23] . |
| | | Compares three case studies to examine experiences of CHWs in efforts to improve access to care through community participation/outreach services. Finds strengthened institutional contexts needed.[24] |
| | | Compares CHW programmes, finds investment in resources, training and support is needed [25]. |
| | | Summarizes key features of CHW programme and response to HIV/AIDS.[26] |
| | Sub-Saharan Africa | Compares ´hidden´ community/village level volunteers with formal, paid CHWs, finds need to recognise hidden volunteers.[27] |
| | Tanzania | Community Health Systems Strengthening (CHSS) model utilises formal/informal networks in communities to address gaps in services. Can support and legitimise CTC (close-to-community) providers and create sustainable community-based programmes. [21] |
| | Zambia | Examines appropriate incentive package for provision of care at community level and argues CHW Programme Development and Implementation Committee should be established.[28] |
| | LMICs | Examines various incentives to motivate and retain CHWs, recommends more systematic use of multiple incentives, emphasizes importance of relationships between CHW and community.[29] |
| | | Examines growth, geographical distribution and programmatic orientations of literature on CHWs over 10 years. [30] |
| **Committees (>10 studies)** | Asia | Finds community participation (through community health structures, decentralization, community financing) needs more investment by the state, stronger evidence. [31] |
| | Bangladesh | Health Watch Committees improved community health service awareness/advocated for better service provision, hindered by lack of legal accountability/authority.[32] |
| | Kenya | Leaders should be nurtured across governance structures to improve resilience in health systems. [33] |
| | | Examines facility management committees, highlighting feasibility and challenges of engaging community in health planning process. [34] |
| | Nigeria | Community health committees found to be strong support for PHC. [35] |
| | South Africa | Overview of health committee functioning and recommendations going forward, including identifying capacity and training needs.[36] |
| | | Leaders should be nurtured across governance structures to improve resilience in health systems. [33] |
| | | Explores relationship between participation and right to health, lessons of best practice for community participation from health committees: balance of power, intersectoral activity, apprenticeship, link between action and change, use of sources of information. [37] |
| | | Describes three-year health committee intervention and critical factors for enhancing their potential to drive community participation. [38] |
| | Tanzania | Explores views of villagers on PHC committees, village health workers, skills staff and responsiveness to community health needs, finds more regular feedback on health service delivery constraints and existing community-based health organisations is needed for participation. [39] |
| | Zambia | Examines effect of HIV service scale-up on mechanisms of accountability in primary health facilities, calls for greater research/understanding. [40] |
| | LMICS | Narrative review to understand contextual features relevant to committees, develops contextual framework of context (community, health facilities, health administration, society) and cross-cutting issues e.g. trust, awareness, benefits, resources etc. [41] |
| | | Addresses gap between external accountability and bureaucratic accountability mechanism and interactions between them. [9] |
| | | Systematic literature review on evidence on health facility committees´ effectiveness and factors that influence performance/effectiveness. [42] |
| | Zimbabwe | Explores relationship between Health Centre Committees, finds they lead to improved health outcomes/PHC services, but weak formal recognition, poorly resourced/trained, no influence on health budgets.[43] |

| | | |
|---|---|---|
| | Uganda | Describes three-year health committee intervention and critical factors for enhancing their potential to drive community participation. [38] |
| **Community-based monitoring (>10 studies)** | Bangladesh | Explores Community Groups (CGs), finds effective community participation requires individual and community empowerment. CGs are functional but constrained by many factors (bias member selection, lack of official recognition, poor leadership/authority).[44] |
| | Guatemala | Analyses social participation from perspective of power relations in historical, social, economic context of Guatemala.[45] |
| | India | Assesses functionality of National Rural Health Mission (NRHM) in terms of Community-Based Monitoring, which needs to be institutionalized on a larger scale.[46] |
| | | Evaluates community monitoring program, challenges include limited representation, lack of involvement and no chairperson/convenor. Finds need for evaluation framework in planning.[47] |
| | | Literature review on social autopsy (social, behavioural, health systems contributors) of maternal/child deaths, explores Maternal and Perinatal Death Inquiry and Response program. Finds social autopsy powerful for raising awareness, providing evidence, motivating action.[48] |
| | | Examines framework for community-based monitoring and improvement of local health services and limitations. Suggests it is accepted as an accountability principle at all levels of governance.[49] |
| | | Explores power relationships and ethical dilemmas when developing community monitoring systems, highlighting considerations (meanings of autonomy/consent, documentation for transparency, minimizing risks to individuals).[50] |
| | | Examines effectiveness of social audit as accountability tool and impact on implementation of National Rural Employment Guarantee Scheme.[51] |
| | Kenya | Reviews evidence on literature/secondary evidence on community participation, including community voice, district functionality, wider contexts/processes.[52] |
| | Uganda | Randomized field experiment on community-based monitoring of public primary healthcare providers, finding increases in utilization and improved health outcomes.[53] |
| | Zambia | Reviews evidence on literature/secondary evidence on community participation, including community voice, district functionality, wider contexts/processes.[52] |
| | Zimbabwe | Focuses on progress and challenges in health equity, finding weak monitoring and social accountability.[54] |
| | LMICS | Theory-driven review of collective citizen engagement/advocacy cases, insight into perspectives, reasoning, agency, abilities of health providers to respond to citizens. Must evaluate intermediate effects (attitudinal/behavioural changes or social accountability initiatives).[13] |
| **Complaints (<5 studies)** | South Africa | National Guideline to Manage Complaints, Compliments and Suggestions in the Public Health Sector of South Africa: Based on the Patients´ Rights Charter, guidelines/standards monitor whether health facilities adhere to this.[55] |
| | Vietnam | Investigates patents´ complaint handling processes and main influences on their implementation in public hospitals. Proposes policy implications for improvement (improving service provider accountability/better utilisation of information on complaints).[56] |
| | LMICs | Theory-driven review of collective citizen engagement/advocacy cases, insight into perspectives, reasoning, agency, abilities of health providers to responds to citizens. Must evaluate intermediate effects (attitudinal/behavioural changes or social accountability initiatives).[13] |
| | | Addresses gap between external accountability and bureaucratic accountability mechanism and interactions between them.[9] |
| **Discreet choice experiment (<5 studies)** | Liberia | DCE designed to assess preferences for structure and process of care at health clinics. Choice of clinic most influenced by provision of thorough physical exam and consistent available medicine. Respectful treatment and government management played a role.[57] |
| | Tanzania | DCE used to investigate women´s preferences for places of delivery of care. Greatest predictor of health facility preference was kind treatment by a doctor, followed by a doctor with excellent medical knowledge, followed by modern medical equipment and drugs.[58] |
| **Exit interviews (>10 studies)** | India | Assessing users´ and providers´ perspectives in challenges faced in the provision of quality care.[59] |
| | Ghana | Describing provider behaviour related to supply of health services to insured clients in Ghana and the influence of provider payment methods on incentives and behaviour.[60] |
| | Lao PDR | Comparing health system responsiveness between two hospitals.[61] |
| | Sierra Leone | Understanding the factors that influence the selection of a healthcare provider once the decision to seek care has been made, considering cost, location and reputation.[62] |
| | South Africa | Determining patient satisfaction.[63] |
| | Zambia | Exploring how users and providers perceive low utilization of health facilities.[64] |

| | | |
|---|---|---|
| **Human/patient rights (<10 studies)** | LMICs | Explores evidence on community accountability mechanisms, finding not enough empirical data and future studies needed.[65] |
| | India | Citizens´ Charter in Government of India lets people know mandate of Ministry/Department/Organisation, how to get in touch with its officials, what to expect from services and how to seek a remedy if something goes wrong.[66] |
| | Kenya | Examines experiences of health facility charter and awareness of it, with challenges including non-adherence to charter provisions by health workers, illegibility/language issues, lack of expenditure records, no time to read or understand them, socio-cultural limitations.[67] |
| | South Africa | Highlights key issues that constitute/affect health law in post-apartheid South Africa, examining the health system from a rights perspective and making recommendations for future policy and legislative development.[68] |
| | | Explores if human rights paradigm can create space for civil society action, arguing human rights provide a means to contest globalisation constraints.[69] |
| | Uganda | Assess levels of awareness, responsiveness, practice of Uganda Patients´ Charter among patients and health workers, finding limitations.[70] |
| **Information systems (<10 studies)** | India | Assess My Health, My Voice project – technology used to monitor/display online data regarding informal payments for maternal health care, including hotline where women could report health providers´ demands for informal payments. Enhanced knowledge of entitlements, confidence to claim rights.[71] |
| | | Assessed use of ICT in health sector including potential for further use. Findings include Health Management Information Systems, data collection by frontline health workers, community feedback systems, ICT-based education and skill development for healthcare providers, decision-making systems and changing the behaviour of end-users.[72] |
| | Indonesia | Details Expanding Maternal and National Survival (EMAS) project, an SMS and web-based system used to capture, analyse and address citizen feedback.[73] |
| | South Africa | Reviews role of mobile phone technology for monitoring and evaluation of community-based health services, finds insufficient evidence and challenges in implementation and a need for a systems perspective that does not separate technology from its implementation environment.[74] |
| | | Uses Mxit as mobile phone-based social media network to encourage comments on proposed NHI and raise awareness on rights to free and quality healthcare.[75] |
| | LMICs | Reviews IS research and benefits from ICTs, highlighting key themes (failure, outsourcing, strategic value, socio-economic contexts).[76] |
| **Call centre / hotlines / SMS hotlines (<10 studies)** | Bangladesh | Assesses existing evidence on patient complaints management systems and provides practical options for future policy and practice, identifies key outstanding gaps in existing literature. Finds need for comprehensive, integrated, context-specific systems that addresses unequal power relations and information asymmetry.[77] |
| | Burkina Faso | Evaluates a toll-free call service and interactive voice server in improving health system governance. Functional but may be negatively impacted by cultural context, fear or reprisal.[78] |
| | India | Asses My Health, My Voice project – technology used to monitor/display online data regarding informal payments for maternal health care, including hotline where women could report health providers´ demands for informal payments. Enhanced knowledge of entitlements, confidence to claim rights.[71] |
| | South Africa | Analysed feedback through MomConnect, mHealth initiative giving pregnant women information via SMS. 74% of all complaints resolved.[79] |
| | Uganda | Reports on two SMS-based platforms to generate real-time information from citizens/health providers, providing evidence on health service delivery.[80] |
| | Vietnam | Investigates patents´ complaint handling processes and main influences on their implementation in public hospitals. Proposes policy implications for improvement (improving service provider accountability/better utilisation of information on complaints).[56] |
| **Legal (<10 studies)** | LMICs | Assesses social accountability approaches in human development, including national-level legal frameworks providing for access to information.[81] |
| | East & Southern Africa & South Africa | Explores if human rights paradigm can create space for civil society action, arguing human rights provide a means to contest globalisation constraints.[69] |
| | Kenya | Evaluates integration of legal literacy and legal services into healthcare, finding increase in knowledge and awareness.[82] |
| **NGO (>10 studies)** | Ecuador | Explores how an NGO and its health services are perceived by population it services and contributions to reducing barriers to care. Finds positive perceptions but unrealistic expectations at time.[83] |

| | | |
|---|---|---|
| | Kenya | Documents contributions of NGO sector to Kenya´s health goals with potential for higher levels of collaboration.[84] |
| | Mozambique | Reviews evidence on literature/secondary evidence on community participation, including community voice, district functionality, wider contexts/processes.[52] |
| | Myanmar | Community Feedback and Response Mechanism (CFRM) delivers mechanism for community feedback and seek responses in relation to UNDP and other development activities. Promotes accountability.[85] |
| | South Africa | Explores Advocacy, Communication and Social Mobilization (ACSM) Working Group of the Stop TB Partnership to mobilize political, social and financial resources, sustain/expand global movement to eliminate TB, foster development of effective programming.[86] |
| | | Summarizes experiences and results of Treatment Action Campaign (TAC), which mobilized people to campaign for the right to health using human rights education, HIV treatment literacy, demonstration and litigation, with significant results.[87] |
| | Southern Africa | Evaluates civil service organisations (CSOs) in improving HIV prevention efforts at community level with recommendations.[88] |
| | Uganda | Examines case for donors providing financial incentives to NGOs to increase community participation. Finds higher community participation consistent even with reduced beneficiary welfare.[89] |
| | LMICs | Investigates practice of nutrition advocacy and suggests ways to strengthen capacities/practices in the future through three case studies.[90] |
| | LMICs | Theory-driven review of collective citizen engagement/advocacy cases, insight into perspectives, reasoning, agency, abilities of health providers to respond to citizens. Must evaluate intermediate effects (attitudinal/behavioural changes or social accountability initiatives).[13] |
| **Patient advocate/ expert patient (<10 studies)** | Malawi | Expert patients trained to assist with HIV clinic tasks studies, showing they add value to ART services.[91] |
| | South Africa | Examines access to medicines (ATM) context supply/demand barriers from provider perspectives (availability, accessibility, accommodation, acceptability, affordability).[92] |
| **Report cards (<10 studies)** | LMICs | Examines universal design options for report cards, summarizes evidence base, presents LMIC examples, reviews challenges, outlines implementation steps.[93] |
| | | Assesses social accountability approaches in human development, including report cards [81]. |
| | | Explores evidence on community accountability mechanisms, finding not enough empirical data and future studies needed.[94] |
| | Tajikistan | Reports on results from focus groups/key informant interviews with regards to three initial considerations for developing a report card initiative for primary health care (selecting indicators for report card, collecting data, working with existing institutions/stakeholders).[95] |
| **Scorecard (<10 studies)** | Afghanistan | Assesses community scorecards (CSC) feasibility through joint engagement of service providers/community members in design of patient-centred services, assesses impact on service delivery/perceived quality of care. Finds skilled facilitators needed.[96] |
| | Congo | Describes implementation of community scorecards, challenges include transparency, community participation, improved quality of care. Findings are positive, users and providers able to work together to develop solutions.[97] |
| | Ghana | Uses scorecards to access and improve maternal/newborn health services and effectiveness of engaging multiple stakeholders. Shows improvements in accountability, community participation, transparency, clarity of lines of accountability among decision-makers.[98] |
| | Malawi | Reviews experience with Community Score Card, finding contributions to citizen empowerment, service provider and power-holder effectiveness, accountability, responsiveness, spaces of negotiation.[99] |
| | | Reviews evidence on literature/secondary evidence on community participation, including community voice, district functionality, wider contexts/processes.[52] |
| | Tajikistan | Reports on results from focus groups/key informant interviews with regards to three initial considerations for developing a report card initiative for primary health care (selecting indicators for report card, collecting data, working with existing institutions/stakeholders).[95] |
| **Survey/questionnaire (>10 studies)** | Nigeria | Uses out-patient questionnaire from WHO responsiveness survey to evaluate NHIS. Autonym, communication, prompt attention are priority areas for improving responsiveness.[100] |
| | | Household data combined with other data to estimate demand for outpatient health care.[101] |
| | | Measures responsiveness in private/public hospitals, comparing performance to determine impact/relevance for public health.[102] |

| | | |
|---|---|---|
| | Indonesia | Surveys patients on satisfaction, finding continuity of provider, waiting time, availability of amenities, cost and social interaction with provider at bottom of the list.[103] |
| | Tanzania | Studies health system responsiveness to examine relationship with patient factors and visit non-adherence, finds more evidence needed.[104] |
| | | Surveys health system responsiveness in private clinics serving HIV patients. Finds high levels of satisfaction. Confidentiality, communication, respect highly rated.[105] |
| | | Studies patient satisfaction in the out-patient department, finds overall dissatisfaction on quality of care.[106] |
| | South Africa | Describes economic framework for analysis/planning of health system reform to achieve productivity/responsiveness.[107] |
| | | Population-based survey conducted based on WHO health system performance assessment, identifies health care access, communication, autonomy, discriminatory experiences as priority areas.[108] |
| | India | Uses rapid assessment technique in micro-level planning for primary health services, collecting household-level data to estimate client needs, coverage of services and unmet needs to formulate micro-level plans aimed at improving service coverage and quality.[109] |
| | | Surveys family caregivers of hospitalized psychiatrically ill to explore perceived importance of various aspects of interactions, finds provision of informational inputs and addressing of concerns raised as priority areas.[110] |
| | | Explores concept of patient-physician trust and patient satisfaction through descriptive household survey. Finds trust influences patient´s self-reported satisfaction and is independent of other factors assessed in study.[111] |
| | Global /LMIC comparison | Describes WHO study as common survey instrument in nationally representative populations with modular structure for assessing health of individuals in various domains, health system responsiveness, household health care expenditures, additional modules.[112] |
| | | Uses data from World Health Survey to assess individual preferences for prioritizing reductions in health/health inequalities in primary health system goal. Finds individuals prioritize health system goals related to overall improvement.[113] |
| | | Assesses nature, strengths, limitations of treatment gap and resource availability measures that are currently used to assess adequacy of epilepsy care and applicability of WHO new measures. Finds WHO measures conceptually superior but requires data not yet available.[114] |
| | | Theory-driven review of collective citizen engagement/advocacy cases, insight into perspectives, reasoning, agency, abilities of health providers to responds to citizens. Must evaluate intermediate effects (attitudinal/behavioural changes or social accountability initiatives).[13] |
| **Suggestion boxes (<10 studies)** | Myanmar | Community Feedback and Response Mechanism (CFRM) delivers mechanism for community feedback and seek responses in relation to UNDP and other development activities. Promotes accountability.[85] |
| | Nepal | Researches complaint management systems, finds few complaints by service users, recommends establishment of proper complaints mechanisms.[115] |
| | LMICs | Explores evidence on community accountability mechanisms, finding not enough empirical data and future studies needed.[65] |

**References:**


1. Srivastava A, Gope R, Nair N, et al. Are village health sanitation and nutrition committees fulfilling their roles for decentralised health planning and action? A mixed methods study from rural eastern India. *BMC Public Health*. 2015;16(1):59:1-12. doi:10.1186/s12889-016-2699-4.
2. Lodenstein E, Dao D. Devolution and human resources in primary healthcare in rural Mali. *Hum Resour Health*. 2011;9:15;1-6. doi:10.1186/1478-4491-9-15.
3. Oyaya C, Rifkin S. Health Sector Reforms in Kenya: an examination of district level planning. *Health Policy Amst Neth*. 2003;64:113-127. doi:10.1016/S0168-8510(02)00164-1.
4. Birn A-E, Zimmerman S, Garfield R. To decentralize or not to decentralize, is that the question? Nicaraguan health policy under structural adjustment in the 1990s. *Int J Health Serv*. 2000;30(1):111-128. doi:10.2190/C6TB-B16Y-60HV-M3QW.
5. Bossert TJ, Mitchell AD. Health sector decentralization and local decision-making: Decision space, institutional capacities and accountability in Pakistan. *Soc Sci Med 1982*. 2011;72(1):39-48. doi:10.1016/j.socscimed.2010.10.019.
6. Semali IA, Tanner M, de Savigny D. Decentralizing EPI services and prospects for increasing coverage: the case of Tanzania. *Int J Health Plann Manage*. 2005;20(1):21-39.doi:10.1002/hpm.794
7. Mubyazi G, Kamugisha M, Mushi A, Blas E. Implications of decentralization for the control of tropical diseases in Tanzania: a case study of four districts. *Int J Health Plann Manage*. 2004;19(S1):S167-S185. doi:10.1002/hpm.776.
8. Berlan D, Shiffman J. Holding health providers in developing countries accountable to consumers: a synthesis of relevant scholarship. *Health Policy Plan*. 2012;27(4):271-280. doi:10.1093/heapol/czr036.
9. Cleary SM, Molyneux S, Gilson L. Resources, attitudes and culture: an understanding of the factors that influence the functioning of accountability mechanisms in primary health care settings. *BMC Health Serv Res*. 2013;13(1):1-13. doi:10.1186/1472-6963-13-320.

10. Ciccone DK, Vian T, Maurer L, Bradley EH. Linking governance mechanisms to health outcomes: A review of the literature in low- and middle-income countries. *Soc Sci Med.* 2014;117:86-95. doi:10.1016/j.socscimed.2014.07.010.
11. Brinkerhoff DW, Bossert TJ. Health governance: principal–agent linkages and health system strengthening. *Health Policy Plan*. 2014;29(6):685-693. doi:10.1093/heapol/czs132.
12. Gohou V, Ronsmans C, Kacou L, et al. Responsiveness to life-threatening obstetric emergencies in two hospitals in Abidjan, Cote d'Ivoire. *Trop Med Int Health*. 2004;9(3):406-415. doi:10.1111/j.1365-3156.2004.01204.x.
13. Lodenstein E, Dieleman M, Gerretsen B, Broerse JEW. Health provider responsiveness to social accountability initiatives in low- and middle-income countries: a realist review. *Health Policy Plan*. 2017;32(1):125-140. doi:10.1093/heapol/czw089.
14. Peters DH, Kanjilal B. Health insurance & responsiveness to communities & patients: The future of health systems in India. *Indian J Med Res*. 2011;133(1):9-10. https://www-ncbi-nlm-nih-gov.ezproxy.uct.ac.za/pmc/articles/PMC3100153/
15. Witter S, Garshong B. Something old or something new? Social health insurance in Ghana. *BMC Int Health Hum Rights*. 2009;9(1):20:1-13. doi:10.1186/1472-698X-9-20.
16. Criel B, Waelkens M, Soors W, Devadasan N, Atim C. Community health insurance in developing countries. *Int Encycl Public Health.* 2008;(1):782-791. doi:10.1016/B978-012373960-5.00159-3.
17. Standing H, Chowdhury AMR. Producing effective knowledge agents in a pluralistic environment: What future for community health workers? *Soc Sci Med*. 2008;66(10):2096-2107. doi:10.1016/j.socscimed.2008.01.046.
18. Choudhury N, Ahmed T, Hossain MdI, et al. Community-based management of acute malnutrition in Bangladesh: Feasibility and constraints. *Food Nutr Bull*. 2014;35(2):277-285. doi:10.1177/156482651403500214.
19. Lehmann U, Sanders D. *Community health workers: What do we know about them? The state of the evidence on programmes, activities, costs an impact on health outcomes of using community health workers*. Geneva: World Health Organization; 2007. https://www.hrhresourcecenter.org/node/1587.html. Accessed June 12, 2020.
20. ICF International. *Global Fund-Supported Community Systems Strengthening Programs in Cambodia: Evaluation Report.* United States of America: The Global Fund to Fight AIDS, Tuberculosis and Malaria; 2012.
21. Lunsford SS, Fatta K, Stover KE, Shrestha R. Supporting close-to-community providers through a community health system approach: case examples from Ethiopia and Tanzania. *Hum Resour Health*. 2015;13(1):1-9. doi:10.1186/s12960-015-0006-6.
22. Saprii L, Richards E, Kokho P, Theobald S. Community health workers in rural India: analysing the opportunities and challenges Accredited Social Health Activists (ASHAs) face in realising their multiple roles. *Hum Resour Health*. 2015;13(1):95:1-13. doi:10.1186/s12960-015-0094-3.
23. van Ginneken N, Lewin S, Berridge V. The emergence of community health worker programmes in the late apartheid era in South Africa: An historical analysis. *Soc Sci Med*. 2010;71(6):1110-1118. doi:10.1016/j.socscimed.2010.06.009.
24. Nxumalo NL. *Community health workers, community participation and community level inter-sectoral action: the challenges of implementing primary health care outreach services* [master's thesis]. South Africa: University of Witwatersrand; 2013.
25. Nxumalo N, Goudge J, Thomas L. Outreach services to improve access to health care in South Africa: lessons from three community health worker programmes. *Glob Health Action*. 2013;6(1):19283:1-9. doi:10.3402/gha.v6i0.19283.
26. Schneider H, Hlophe H, van Rensburg D. Community health workers and the response to HIV/AIDS in South Africa: tensions and prospects. *Health Policy Plan*. 2008;23(3):179-187. doi:10.1093/heapol/czn006.
27. Leon N, Sanders D, Van Damme W, et al. The role of 'hidden' community volunteers in community-based health service delivery platforms: examples from sub-Saharan Africa. *Glob Health Action*. 2015;8(1):27214:1-7- doi:10.3402/gha.v8.27214.
28. Sunkutu K, Nampanya-Serpell N. Searching for common ground on incentive packages for community workers and colunteers in Zambia. World Health Organization. https://www.who.int/workforcealliance/knowledge/resources/incentives_zambia/en/. Published 2009. Accessed October 28, 2019.
29. Bhattacharyya K, Winch P, LeBan K, Tien M. *Community health worker incentives and disincentives: How they affect motivation, retention and sustainability.* Virginia: Basic Support for Institutionalizing Child Survival Project (BASICS II), USAID; 2001.
30. Schneider H, Okello D, Lehmann U. The global pendulum swing towards community health workers in low- and middle-income countries: a scoping review of trends, geographical distribution and programmatic orientations, 2005 to 2014. *Hum Resour Health*. 2016;14(65):1-12. doi:10.1186/s12960-016-0163-2.
31. Murthy RK, Klugman B. Service accountability and community participation in the context of health sector reforms in Asia: implications for sexual and reproductive health services. *Health Policy Plan*. 2004;19(suppl_1):i78-i86. doi:10.1093/heapol/czh048.
32. Barrett G. Community-managed clinics and health watch committees (Bangladesh). Participedia. https://participedia.net/case/16. Published 2010. Accessed October 24, 2019.
33. Gilson L, Barasa E, Nxumalo N, et al. Everyday resilience in district health systems: emerging insights from the front lines in Kenya and South Africa. *BMJ Glob Health*. 2017;2(2):1-15. doi:10.1136/bmjgh-2016-000224.
34. O'Meara WP, Tsofa B, Molyneux S, Goodman C, McKenzie FE. Community and facility-level engagement in planning and budgeting for the government health sector – A district perspective from Kenya. *Health Policy Amst Neth*. 2011;99(3):234-243. doi:10.1016/j.healthpol.2010.08.027.
35. Abimbola S, Negin J, Jan S, Martiniuk A. Towards people-centred health systems: a multi-level framework for analysing primary health care governance in low- and middle-income countries. *Health Policy Plan*. 2014;29(suppl 2):ii29-ii39. doi:10.1093/heapol/czu069.

36. Haricharan H. *Extending participation: Challenges of health committees as meaningful structures for community participation*. South Africa: Human Rights Division, School of Public Health, University of Cape Town and The Learning Network on Health and Human Rights; 2010.
37. Glattstein-Young G, London L. Community health committees as a vehicle for participation in advancing the right to health. *Crit Health Perspect*. 2010;2:1-2.
38. Mulumba M, London L, Nantaba J, Ngwena C. Using health committees to promote community participation as a social determinant of the right to health: Lessons from Uganda and South Africa. *Health Hum Rights*. 2018;20(2):11-17. PMC6293345
39. Mubyazi GM. Local primary health care committees and community-based health workers in Mkuranga District, Tanzania: Does the public recognise and appreciate them? *Stud Ethno-Med*. 2007;1(1):27-35. doi:10.31901/24566772.2007/01.01.03.
40. Topp SM, Black J, Morrow M, Chipukuma JM, Van Damme W. The impact of human immunodeficiency virus (HIV) service scale-up on mechanisms of accountability in Zambian primary health centres: a case-based health systems analysis. *BMC Health Serv Res*. 2015;15(1):1-14. doi:10.1186/s12913-015-0703-9.
41. George A, Scott K, Garimella S, Mondal S, Ved R, Sheikh K. Anchoring contextual analysis in health policy and systems research: A narrative review of contextual factors influencing health committees in low and middle income countries. *Soc Sci Med*. 2015;133:159-167. doi: 10.1016/j.socscimed.2015.03.049
42. McCoy DC, Hall JA, Ridge M. A systematic review of the literature for evidence on health facility committees in low- and middle-income countries. *Health Policy Plan*. 2012;27(6):449-466. doi:10.1093/heapol/czr077.
43. Loewenson R, Rusike I, Zulu M. *The impact of health centre committees on health outcomes in Zimbabwe. Paper Presented to Forum 9, Global Forum for Health Research*. South Africa: Southern African Regional Network on Equity in Health (EQUINET); 2005.
44. Mahmud S. Citizen participation in the health sector in rural Bangladesh: Perceptions and reality. *IDS Bull*. 2004;35(2):11-18. doi:10.1111/j.1759-5436.2004.tb00116.x.
45. Flores W, Ruano AL, Funchal DP. Social participation within a context of political violence: Implications for the promotion and exercise of the right to health in Guatemala. *Health Hum Rights*. 2009;11(1):37-48. doi:10.2307/40285216.
46. Garg S, Laskar AR. Community-based monitoring: Key to success of national health programs. *Indian J Community Med Off Publ Indian Assoc Prev Soc Med*. 2010;35(2):214-216. doi:10.4103/0970-0218.66857.
47. Tripathy JP, Aggarwal AK, Patro BK, Verma H. Process evaluation of community monitoring under national health mission at Chandigarh, union territory: Methodology and challenges. *J Fam Med Prim Care*. 2015;4(4):539-45s. doi:10.4103/2249-4863.174282.
48. Kalter HD, Salgado R, Babille M, Koffi AK, Black RE. Social autopsy for maternal and child deaths: a comprehensive literature review to examine the concept and the development of the method. *Popul Health Metr*. 2011;9:45:1-13. doi:10.1186/1478-7954-9-45.
49. Kakade D. Community-based monitoring as an accountability tool: influence on rural health services in Maharashtra, India. *BMC Proc*. 2012;6(Suppl 1):1-2. doi:10.1186/1753-6561-6-S1-O9.
50. Khanna R. *Ethical issues in community based monitoring of health programmes: Reflections from India*. Society for Health Alternatives India (SAHAJ) with Community of Practitioners on Accountability and Social Action in Health (COPASAH). India: Centre for Health and Social Justice; 2013.
51. Singh R, Vutukuru V. Enhancing accountability in public service delivery through social audits: A case study of Andhra Pradesh, India. Namati. https://namati.org/resources/enhancing-accountability-in-public-service-delivery-through-social-audits-a-case-study-of-andhra-pradesh-india/. Published 2010. Accessed October 25, 2019.
52. Baez C, Barron P. *Community Voice and Role in District Health Systems in East and Southern Africa: A Literature Review. EQUINET Discussion Paper 39.* South Africa: Regional Network for Equity in Health in East and Southern Africa (EQUINET); 2006.
53. Björkman M, Svensson J. Power to the people: evidence from a randomized field experiment on community-based monitoring in Uganda. *Q J Econ*. 2009;(124):735-769. doi.org/10.1162/qjec.2009.124.2.735
54. Training and Research Centre and Ministry of Health and Child Care. *Zimbabwe Equity Watch 2014*. Harare: TARSC, MoHCC, EQUINET; 2014.
55. South African Department of Health. *National Guideline to Manage Complaints, Compliments and Suggestions in the Public Health Sector of South Africa*. South Africa: South African Department of Health; 2017..
56. Thi Thu Ha B, Mirzoev T, Morgan R. Patient complaints in healthcare services in Vietnam's health system. *SAGE Open Med*. 2015;3:1-9. doi:10.1177/2050312115610127
57. Kruk ME, Rockers PC, Varpilah ST, Macauley R. Population preferences for health care in Liberia: Insights for rebuilding a health system. *Health Serv Res*. 2011;46(6):1-22. doi:10.1111/j.1475-6773.2011.01266.x.
58. Larson E, Vail D, Mbaruku GM, Kimweri A, Freedman LP, Kruk ME. Moving toward patient-centered care in Africa: A discrete choice experiment of preferences for delivery care among 3,003 Tanzanian women. Dalal K, ed. *PLOS ONE*. 2015;10(8):1-12. doi:10.1371/journal.pone.0135621.
59. Bhattacharyya S, Issac A, Rajbangshi P, Srivastava A, Avan BI. "Neither we are satisfied nor they"-users and provider's perspective: a qualitative study of maternity care in secondary level public health facilities, Uttar Pradesh, India. *BMC Health Serv Res*. 2015;15(1):1-13. doi:10.1186/s12913-015-1077-8.
60. Agyepong IA, Aryeetey GC, Nonvignon J, et al. Advancing the application of systems thinking in health: provider payment and service supply behaviour and incentives in the Ghana National Health Insurance Scheme – a systems approach. *Health Res Policy Syst*. 2014;12:1-18. doi:10.1186/1478-4505-12-35.
61. Douangvichit D, Liabsuetrakul T. Obstetric care and health system responsiveness for hospital-based delivery in Lao People's Democratic Republic. *J Med Assoc Thai*. 2012;95(9):1126-1135. https://pubmed.ncbi.nlm.nih.gov/23140028/

62. Jacobsen KH, Ansumana R, Abdirahman HA, et al. Considerations in the selection of healthcare providers for mothers and children in Bo, Sierra Leone: reputation, cost and location. *Int Health*. 2012;4(4):307-313. doi:10.1016/j.inhe.2012.09.004.
63. Chimbindi N, Bärnighausen T, Newell M-L. Patient satisfaction with HIV and TB treatment in a public programme in rural KwaZulu-Natal: evidence from patient-exit interviews. *BMC Health Serv Res*. 2014;14:32:1-13. doi:10.1186/1472-6963-14-32.
64. Phiri SN, Fylkesnes K, Ruano AL, Moland KM. 'Born before arrival': user and provider perspectives on health facility childbirths in Kapiri Mposhi district, Zambia. *BMC Pregnancy Childbirth*. 2014;14(1):1-10. doi:10.1186/1471-2393-14-323.
65. Molyneux S, Atela M, Angwenyi V, Goodman C. Community accountability at peripheral health facilities: a review of the empirical literature and development of a conceptual framework. *Health Policy Plan*. 2012;27(7):541-554. doi:10.1093/heapol/czr083.
66. Department of Administrative Reforms and Public Grievances, Government of India. Citizens´ Charters in Government of India. Government of India. https://www.goicharters.nic.in/. Published 2019. Accessed July 10, 2019.
67. Atela M, Bakibinga P, Ettarh R, Kyobutungi C, Cohn S. Strengthening health system governance using health facility service charters: a mixed methods assessment of community experiences and perceptions in a district in Kenya. *BMC Health Serv Res*. 2015;15(1):539:1-12. doi:10.1186/s12913-015-1204-6.
68. Hassim A, Heywood M, Berger J. *Health & Democracy: A Guide to Human Rights, Health Law and Policy in Post-Apartheid South Africa*. South Africa: SiberInk Cape Town; 2007.
69. London L, Schneider H. Globalisation and health inequalities: Can a human rights paradigm create space for civil society action? *Soc Sci Med*. 2012;74(1):6-13. doi:10.1016/j.socscimed.2011.03.022.
70. Kagoya HR, Kibuule D, Mitonga-Kabwebwe H, Ekirapa-Kiracho E, Ssempebwa JC. Awareness of, responsiveness to and practice of patients' rights at Uganda's national referral hospital. *Afr J Prim Health Care Fam Med*. 2013;5(1):1-7. doi:10.4102/phcfm.v5i1.491.
71. Dasgupta J, Sandhya YK, Lobis S, Verma P, Schaaf M. Using technology to claim rights to free maternal health care: Lessons about impact from the My Health, My Voice pilot project in India. *Health Hum Rights*. 2015;17(2,):135-147. https://pubmed.ncbi.nlm.nih.gov/26766855/
72. Garai A, Ganesan R. Role of information and communication technologies in accelerating the adoption of healthy behaviors. Published 2010. https://papers.ssrn.com/abstract=1943597. Accessed October 7, 2019.
73. International Telecommunications Union. Citizen feedback mechanisms. ITU. Published 2019. http://emasindonesia.org. Accessed July 5, 2019.
74. Leon N, Schneider H. *MHealth4CBS in South Africa: A review of the role of mobile phone technology for the monitoring and evaluation of community based health services*. Cape Town: Medical Research Council and University of Western Cape; 2012.
75. Weimann E, Stuttaford MC. Consumers' perspectives on National Health Insurance in South Africa: Using a mobile health approach. *JMIR MHealth UHealth*. 2014;2(4):1-14. doi:10.2196/mhealth.3533.
76. Avgerou C. Information systems in developing countries: a critical research review. *J Inf Technol*. 2008;23(3):133-146. doi:10.1057/palgrave.jit.2000136.
77. Mirzoev T, Kane S. Key strategies to improve systems for managing patient complaints within health facilities – what can we learn from the existing literature? *Glob Health Action*. 2018;11(1):1-14. doi:10.1080/16549716.2018.1458938.
78. Lechat L, Bonnet E, Queuille L, Traoré Z, Somé P-A, Ridde V. Relevance of a toll-free call service using an interactive voice server to strengthen health system governance and responsiveness in Burkina Faso. *Int J Health Policy Manag*. 2019;8(6):353-364. doi:10.15171/ijhpm.2019.13.
79. Barron P, Pillay Y, Fernandes A, Sebidi J, Allen R. The MomConnect mHealth initiative in South Africa: Early impact on the supply side of MCH services. *J Public Health Policy*. 2016;37(2):201-212. doi:10.1057/s41271-016-0015-2.
80. Nkrumah Y, Mensah J, Bujoreanu L. *Crowdsourcing feedback to improve healthcare systems*. Washington (DC): The World Bank; 2014.
81. Ringold D, Holla A, Koziol M, Srinivasan S. *Citizens and service delivery : Assessing the use of social accountability approaches in the human development sectors*. Washington (DC): The World Bank; 2012.
82. Gruskin S, Safreed-Harmon K, Ezer T, Gathumbi A, Cohen J, Kameri-Mbote P. Access to justice: evaluating law, health and human rights programmes in Kenya. *J Int AIDS Soc*. 2013;16:1-7. doi:10.7448/IAS.16.3.18726.
83. Biermann O, Eckhardt M, Carlfjord S, Falk M, Forsberg BC. Collaboration between non-governmental organizations and public services in health – a qualitative case study from rural Ecuador. *Glob Health Action*. 2016;9(1):1-12. doi:10.3402/gha.v9.32237.
84. Berman P, Et al. *Kenya: Non-governmental health care provision. Data for decision making project.* United States of America: Department of Population and International Health, Harvard School of Public Health, and African Medical Research Foundation (AMREF); 1995.
85. Decision Support Service Co., Ltd. *Community Feedback and Response Mechanism (CFRM)*. Myanmar: Monitoring & Evaluation Unit UNDP Yangon; 2013.
86. Deane J, Parks W, Stop TB Partnership (World Health Organization). *Advocacy, communication and social mobilization to fight TB: A 10-year framework for action*. Geneva: World Health Organization, Stop TB Partnership; 2006.
87. Heywood M. South Africa's Treatment Action Campaign: Combining law and social mobilization to realize the right to health. *J Hum Rights Pract*. 2009;1(1):14-36. doi:10.1093/jhuman/hun006.
88. Kelly K, Rau A, Stern E. *Community entry points: Opportunities and strategies for engaging community supported HIV/AIDS prevention responses*. Geneva: Global Fund; 2010.
89. Burger R, Dasgupta I, Owens T. Why pay NGOs to involve the community? *Ann Public Coop Econ*. 2015;86(1):7-31. doi:10.1111/apce.12065.

90. Pelletier D, Haider R, Hajeebhoy N, Mangasaryan N, Mwadime R, Sarkar S. The principles and practices of nutrition advocacy: Evidence, experience and the way forward for stunting reduction: Principles and practices of nutrition advocacy. *Matern Child Nutr*. 2013;9:83-100. doi:10.1111/mcn.12081.
91. Tenthani L, Cataldo F, Chan AK, Bedell R, Martiniuk AL, Lettow M van. Involving expert patients in antiretroviral treatment provision in a tertiary referral hospital HIV clinic in Malawi. *BMC Health Serv Res*. 2012;12(1):1-8. doi:10.1186/1472-6963-12-140.
92. Magadzire BP, Budden A, Ward K, Jeffery R, Sanders D. Frontline health workers as brokers: provider perceptions, experiences and mitigating strategies to improve access to essential medicines in South Africa. *BMC Health Serv Res*. 2014;14(1):1-10. doi:10.1186/s12913-014-0520-6.
93. Mcnamara P. Provider-specific report cards: a tool for health sector accountability in developing countries. *Health Policy Plan*. 2006;21(2):101-109. doi:10.1093/heapol/czj009.
94. Molyneux S, Atela M, Angwenyi V, Goodman C. Community accountability at peripheral health facilities: a review of the empirical literature and development of a conceptual framework. *Health Policy Plan*. 2012;27(7):541-554. doi:10.1093/heapol/czr083.
95. Bauhoff S, Tkacheva O, Rabinovich L, Bogdan O. Developing citizen report cards for primary care: evidence from qualitative research in rural Tajikistan. *Health Policy Plan*. 2016;31(2):259-266. doi:10.1093/heapol/czv052.
96. Edward A, Osei-Bonsu K, Branchini C, Yarghal T, Arwal SH, Naeem AJ. Enhancing governance and health system accountability for people centered healthcare: an exploratory study of community scorecards in Afghanistan. *BMC Health Serv Res*. 2015;15(1):1-15. doi:10.1186/s12913-015-0946-5.
97. Ho LS, Labrecque G, Batonon I, Salsi V, Ratnayake R. Effects of a community scorecard on improving the local health system in Eastern Democratic Republic of Congo: qualitative evidence using the most significant change technique. *Confl Health*. 2015;9:1-11. doi:10.1186/s13031-015-0055-4.
98. Blake C, Annorbah-Sarpei NA, Bailey C, et al. Scorecards and social accountability for improved maternal and newborn health services: A pilot in the Ashanti and Volta regions of Ghana. *Int J Gynecol Obstet*. 2016;135(3):372-379. doi:10.1016/j.ijgo.2016.10.004.
99. Gullo S, Galavotti C, Altman L. A review of CARE's Community Score Card experience and evidence. *Health Policy Plan*. 2016;31(10):1-10. doi:10.1093/heapol/czw064.
100. Mohammed S, Bermejo JL, Souares A, Sauerborn R, Dong H. Assessing responsiveness of health care services within a health insurance scheme in Nigeria: users' perspectives. *BMC Health Serv Res*. 2013;13(1):1-13. doi:10.1186/1472-6963-13-502.
101. Akin JS, Guilkey DK, Hazel^Denton E. Quality of services and demand for health care in Nigeria: A multinomial probit estimation. *Soc Sci Med*. 1995;40(11):1527-1537. doi:10.1016/0277-9536(94)00274-W.
102. Adesanya T, Gbolahan O, Ghannam O, et al. Exploring the responsiveness of public and private hospitals in Lagos, Nigeria. *J Public Health Res*. 2012;1(1):2-6. doi:10.4081/jphr.2012.e2.
103. Bernhart MH, Wiadnyana IGP, Wihardjo H, Pohan I. Patient satisfaction in developing countries. *Soc Sci Med*. 1999;48(8):989-996. doi:10.1016/S0277-9536(98)00376-1.
104. Poles G, Li M, Siril H, et al. Factors associated with different patterns of nonadherence to HIV care in Dar es Salaam, Tanzania. *J Int Assoc Provid AIDS Care JIAPAC*. 2014;13(1):78-84. doi:10.1177/1545109712467068.
105. Miller JS, Mhalu A, Chalamilla G, et al. Patient satisfaction with HIV/AIDS care at private clinics in Dar es Salaam, Tanzania. *AIDS Care*. 2014;26(9):1150-1154. doi:10.1080/09540121.2014.882487.
106. Khamis K, Njau B. Patients' level of satisfaction on quality of health care at Mwananyamala hospital in Dar es Salaam, Tanzania. *BMC Health Serv Res*. 2014;14:1-8 doi:10.1186/1472-6963-14-400.
107. Ruff B, Mzimba M, Hendrie S, Broomberg J. Reflections on health-care reforms in South Africa. *J Public Health Policy*. 2011;32(S1):S184-S192. doi:10.1057/jphp.2011.31.
108. Peltzer K. Patient experiences and health system responsiveness in South Africa. *BMC Health Serv Res*. 2009;9:117:1-12. doi:10.1186/1472-6963-9-117.
109. Satia J, Mavalankar D, Sharma B. Micro-level planning using rapid assessment for primary health care services. *Health Policy Plan*. 1994;9(3):318-330. doi:10.1093/heapol/9.3.318.
110. Dinakaran P, Mehrotra S, Bharath S. Interactional aspects of care during hospitalization: Perspectives of family caregivers of psychiatrically ill in a tertiary care setting in India. *Asian J Psychiatry*. 2014;12:63-68. doi:10.1016/j.ajp.2014.06.013.
111. Baidya M, Gopichandran V, Kosalram K. Patient-physician trust among adults of rural Tamil Nadu: A community-based survey. *J Postgrad Med*. 2014;60(1):21-26. doi:10.4103/0022-3859.128802.
112. Üstün TB, Chatterji S, Villanueva M, Bendib L, Sadana R, Valentine N, et al. WHO multi-country survey study on health and responsiveness 2000-2001. Published 2001. https://www.semanticscholar.org/paper/WHO-Multi-country-Survey-Study-on-Health-and-%C3%9Cst%C3%BCn-Chatterji/5e6db20961c15c0c349ff37d779e5c1d7ac21d2f. Accessed October 1, 2019.
113. King NB, Harper S, Young ME. Who cares about health inequalities? Cross-country evidence from the World Health Survey. *Health Policy Plan*. 2013;28(5):558-571. doi:10.1093/heapol/czs094.
114. Begley CE, Baker GA, Beghi E, Butler J, Chisholm D, Langfitt J, et al. Cross-country measures for monitoring epilepsy care. *Epilepsia*. 2007;48(5):990-1001. doi:10.1111/j.1528-1167.2007.00981.x.
115. Gurung G, Derrett S, Gauld R, Hill PC. Why service users do not complain or have 'voice': a mixed-methods study from Nepal's rural primary health care system. *BMC Health Serv Res*. 2017;17(1):1-10. doi:10.1186/s12913-017-2034-5.
